# A Multi-Sensor Experimental Framework for Assessing Occupant and Vehicle Dynamics in Crash Test Scenarios

**DOI:** 10.3390/s26185828

**Published:** 2026-09-14

**Authors:** Oana-Victoria Stanciuc-Otat, Burkhard Scholz, Ilie Dumitru, Cosmin Berceanu

**Affiliations:** 1 Faculty of Mechanical, University of Craiova, 200512 Craiova, Romania; oana.otat@edu.ucv.ro (O.-V.S.-O.); ilie.dumitru@edu.ucv.ro (I.D.); 2 IAV Fahrzeugsicherheit GmbH & Co. KG, Junkers-Ring 10, 85098 Großmehring, Germany; burkhard.scholz@iav.de

**Keywords:** multi-sensor data acquisition, anthropomorphic test device (ATD), thoracic acceleration, seat belt loading, B-pillar acceleration, crash testing, out-of-position (OOP)

## Abstract

The proposed synchronized multi-sensor framework provides an experimental approach for integrated crash assessment by combining vehicle structural response, restraint-system loading, and occupant biomechanical measurements within a common temporal reference. The experimental campaign comprised two full-scale crash events involving three vehicles and six Hybrid III 50th percentile male ATD exposures: a front-to-rear vehicle collision with standard occupant positioning and a full-frontal rigid-barrier impact with forward-leaning out-of-position (OOP) occupants. Triaxial thoracic accelerations, seat belt forces, and B-pillar accelerations were recorded simultaneously using synchronized data acquisition systems. The frontal OOP configuration produced the highest longitudinal (*X*-axis) thoracic acceleration, approximately 62 g, and the highest measured local belt-segment force, 9.80 kN, whereas the rear-impacted vehicle exhibited the highest local B-pillar acceleration. These configuration-specific observations demonstrate the capability of the proposed framework to characterize structural, restraint-system, and occupant responses simultaneously across different full-scale crash configurations.

## 1. Introduction

The assessment of occupant safety in vehicle crash scenarios has evolved significantly with the advancement of sensor technologies and their integration into anthropomorphic test devices (ATDs) and vehicle structures. Modern crash testing relies on high-resolution, high-frequency measurements to capture the complex interaction between the vehicle, restraint systems, and occupants under various impact conditions. Sensor-based instrumentation enables detailed evaluation of biomechanical responses, contributing to improved injury prediction and safety system optimization.

### 1.1. Sensor Instrumentation of Anthropomorphic Test Devices

The evolution of crash test dummies has been pivotal in advancing automotive safety, providing invaluable insights into the dynamics of vehicle collisions and occupant protection. Since their inception, crash test dummies have undergone significant transformations, evolving from rudimentary models to sophisticated instruments equipped with advanced sensor technology. This progression reflects the growing emphasis on precision and accuracy in safety testing, driven by the need to reduce fatalities and injuries in road accidents.

The development of anthropomorphic test devices (ATDs) has progressively incorporated biomechanically based design requirements to improve the representation and measurement of human responses under impact loading. The Hybrid III 50th percentile male ATD represented an important step in this development, introducing biomechanically based characteristics for occupant response assessment [1]. Subsequent developments have addressed limitations in occupant representation and biofidelity, including the development of additional dummy sizes and specific anthropometries [2], as well as more advanced ATDs such as the THOR 50th percentile male [3].

As crash-test instrumentation requirements evolved, ATDs incorporated increasingly comprehensive sensor systems for measuring biomechanical responses. Anthropomorphic test devices are extensively instrumented with sensors to measure accelerations, forces, moments, and displacements at critical anatomical locations. Among these measurements, chest acceleration has been widely recognized as a key indicator of thoracic injury risk, particularly in frontal and rear-end impact scenarios [4,5]. Accelerometers mounted in the thoracic region of ATDs allow for the evaluation of occupant kinematics and dynamic loading during short-duration, high-severity crash events.

Current ATD models, such as Hybrid III and THOR, incorporate multi-axis accelerometers and load cells to capture detailed biomechanical responses under standardized test conditions [6]. These sensors provide standardized measurements that support comparative studies across different impact configurations and seating positions.

Head displacement of the KPSIT C50 dummy, representing a 50th percentile male, in comparison with the KPSIT C5 dummy, representing a 5th percentile female, under low-speed collision conditions was also investigated [7,8].

### 1.2. Out-of-Position Occupant Scenarios

While conventional crash tests typically assume a standard seating posture, real-world crashes often involve occupants in non-ideal or out-of-position (OOP) configurations. OOP conditions can influence injury mechanisms due to altered occupant kinematics and modified interaction with restraint systems [9]. Sensor-based measurements are particularly valuable in these scenarios, as they enable direct quantification of changes in thoracic acceleration and loading patterns relative to nominal seating positions.

Recent studies have emphasized the importance of evaluating OOP configurations to better understand occupant vulnerability and to improve the robustness of passive safety systems [10,11]. Accelerometer data collected from the chest region of ATDs can be used to characterize changes in occupant response associated with different seating positions.

The influence of non-standard seating configurations on occupant response has been investigated under different impact conditions. In frontal impacts, reclined seating has been shown to alter occupant kinematics and biomechanical loading [12]. Accessory lumbar supports have also been shown to modify the biomechanical response of the Hybrid III 50th percentile male ATD [13]. In rear impacts, seat and head-restraint characteristics can substantially influence head–neck kinematics [14], while severe rear-impact testing with out-of-position occupants has further demonstrated the importance of seatback behavior in the resulting occupant response [15].

### 1.3. Seat Belt Force

The seat belt remains one of the primary restraint systems in modern vehicles, and its interaction with the occupant plays a critical role in controlling occupant motion during a crash. Force transducers installed at selected seat belt segments enable direct measurement of local webbing tension during the impact event [16]. These measurements provide information on restraint-system loading and on the responses of components such as pretensioners and load limiters.

Seat belt loading has a direct influence on thoracic response, and variations in belt force can modify the mechanisms associated with chest injury during frontal impact [17]. Load-limiting strategies have also been investigated as a means of reducing injury risk in new seating positions [18]. For elderly occupants, adaptive load-limiting concepts have been shown to improve chest protection in frontal crashes [19].

### 1.4. Vehicle Structural Acceleration Measurements

In addition to occupant-centric sensing, structural acceleration measurements provide information on the local vehicle response during impact. Accelerometers mounted at structural locations, such as the B-pillar, can be used to record longitudinal (X-direction) accelerations associated with the transient response of the occupant compartment [20]. These measurements complement occupant and restraint-system data by providing a local structural reference within the synchronized crash event.

The B-pillar is an important structural component of the occupant compartment, and acceleration measurements at this location can contribute to the characterization of the local structural response under different impact conditions [21]. When evaluated together with ATD and restraint measurements, these data support a multi-domain description of the crash response without being interpreted as vehicle center-of-gravity acceleration or a direct measure of energy transfer.

### 1.5. Integrated Sensor-Based Approaches

Recent research trends emphasize the integration of multiple sensor data streams to characterize occupant–vehicle interaction during crash events. Combined measurements of seat belt loading and ATD biomechanical response have been used to evaluate the influence of restraint characteristics on occupant response [22]. Frontal impact studies have further employed instrumented Hybrid III ATDs to investigate occupant response under different restraint configurations [23]. Comparative experimental investigations using different occupant surrogates have demonstrated the value of thoracic measurements for characterizing biomechanical response under impact loading [24]. Experimental measurements have also been used as a basis for estimating thoracic response in frontal crash tests, illustrating their potential for the development and evaluation of occupant-response models [25]. The increasing availability of high-bandwidth sensors and synchronized data acquisition systems further enhances the capability to characterize complex crash scenarios with high temporal resolution.

Despite these advances, vehicle structural response, restraint-system loading, and occupant biomechanics are still frequently evaluated as separate measurement domains. Their combined temporal characterization within a synchronized experimental framework therefore remains insufficiently addressed, particularly across different impact configurations and non-standard occupant postures.

Accordingly, the objective of this study is to experimentally characterize occupant–vehicle response using synchronized structural, restraint-system, and ATD measurements in two full-scale crash configurations that are less commonly documented in the open literature. The experimental campaign comprises a car-to-car rear impact with deliberate underride-type engagement and instrumented occupants in both vehicles and a 56 km/h full-frontal rigid-barrier impact with forward-leaning Hybrid III 50th percentile male ATDs and local seat belt force measurements at the B3–B6 positions.

The contribution of the study lies primarily in the experimental characterization of these two less commonly documented full-scale crash configurations through the combined analysis of local vehicle structural acceleration, local seat belt webbing tensions, and ATD thoracic response within a common temporal reference. Synchronization of these measurement domains provides the experimental basis for examining their temporal characteristics consistently across the investigated configurations.

- The main contributions of this study are:
- experimental characterization of a full-scale car-to-car rear impact with deliberate underride-type engagement and instrumented occupants in both vehicles;
- experimental characterization of a 56 km/h full-frontal rigid-barrier impact with forward-leaning Hybrid III 50th percentile male ATDs and B3–B6 seat belt force instrumentation;
- synchronized characterization of local structural acceleration, local belt webbing loading, and occupant thoracic response in these two crash configurations; and
- provision of an experimental basis for further investigation of occupant–vehicle interaction and future computational-model evaluation.

## 2. Materials and Methods

### 2.1. Impact Configurations

The experimental campaign was conducted at the IAV Vehicle Safety facility using the facility’s test equipment, anthropomorphic test devices (ATDs), and sensor systems. It comprised two independent full-scale crash events involving three vehicles and six ATD exposures. The first event was a front-to-rear vehicle collision involving two vehicles, with two ATDs positioned in each vehicle. The second event was a full-frontal rigid-barrier impact involving one vehicle with two ATDs in a forward-leaning out-of-position (OOP) configuration. Each impact configuration was represented by a single full-scale crash event; therefore, measurements obtained from multiple ATDs or vehicles within the same event are treated as synchronized observations from that event rather than as independent experimental replicates. No repeat crash tests were performed for either configuration.

Vehicle make and model are intentionally not disclosed because vehicle-specific safety performance is outside the scope of this study. To provide sufficient context regarding the vehicle and restraint-system generation, the approximate model-year range, vehicle class, body style, and relevant front restraint-system characteristics are summarized in Table 1. The vehicles are used as experimental platforms for the investigated crash configurations, and the analysis is not intended to rank or compare individual vehicle models.

#### 2.1.1. Front–Rear-End Impact Test

**Figure 1:**
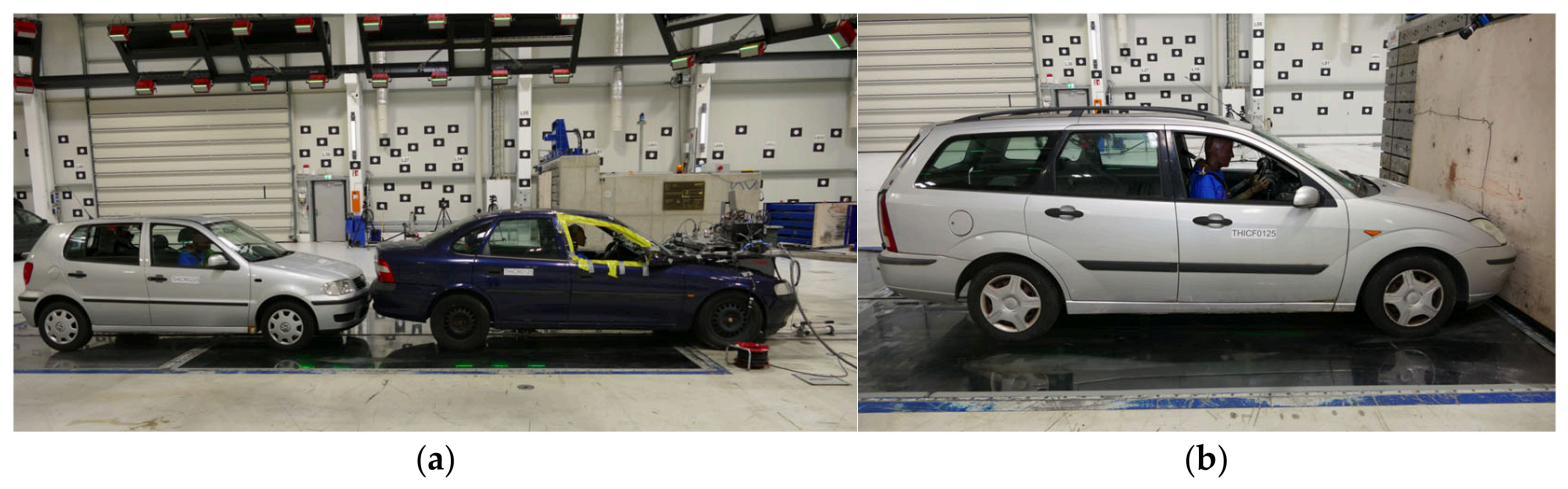
Impact Configurations: (**a**) Front–Rear-End Impact Test; (**b**) Full-Frontal Rigid-Barrier.

The first crash event consisted of a full-overlap front-to-rear collision (Figure 1a) at a relative approach angle of 0°. Both vehicles were instrumented and equipped with two Hybrid III 50th percentile male ATDs in standard front seating positions. The target vehicle was stationary at the time of impact. The track speed of the striking vehicle was set to 56 km/h, while the measured impact speed was 56.11 km/h. Since the target vehicle was stationary, the corresponding closing speed was also 56.11 km/h.

The test mass was 1450 kg for the rear-impacted (target) vehicle and 1220 kg for the striking vehicle. For both vehicles, the reported test mass includes two Hybrid III 50th percentile male ATDs (approximately 78 kg each) and the installed measurement equipment (approximately 40 kg in total).

The vertical bumper alignment was configured to reproduce a realistic rear-impact geometry. At initial contact, the front bumper of the striking vehicle was positioned below the rear bumper of the target vehicle, resulting in an intentional underride-type engagement. The vehicle ride heights were configured to represent the pitch conditions associated with a braking scenario, with rear suspension extension of the target vehicle and front suspension compression of the striking vehicle. Neither vehicle was actively braked at the time of impact.

#### 2.1.2. Full-Frontal Rigid-Barrier Impact with OOP Configuration

The second crash event consisted of a full-frontal impact against a rigid barrier (Figure 1b), with an impact angle of 0°. Two Hybrid III 50th percentile male ATDs were positioned in the front seats in a forward-leaning OOP configuration. The track speed was set to 56 km/h, while the measured vehicle speed at the time of initial contact (t_0_) was 55.90 km/h.

The vehicle test mass was 1480 kg, including two Hybrid III 50th percentile male ATDs (approximately 78 kg each) and the installed measurement equipment (approximately 40 kg in total). No specific adjustment of the vehicle ride height or bumper height was applied; the vehicle remained at the ride height resulting from the defined test loading condition.

The impact configuration was defined with reference to the belted full-frontal rigid-barrier conditions of FMVSS 208. The selected target speed of 56 km/h was higher than the 50 km/h impact speed specified for the Euro NCAP full-width frontal test [26].

For both crash events, the test vehicle was accelerated using the facility track propulsion system. The vehicle was connected to the propulsion system at the front axle, and the target speed and vehicle test mass were specified as input parameters. Based on the defined test configuration, the required starting position along the test track was determined to achieve the prescribed impact speed. Approximately 1 m before the impact point, the vehicle was automatically decoupled from the propulsion system, allowing it to travel freely during the final approach and at the moment of impact.

In both scenarios, synchronized measurements were performed at three levels of interest: vehicle structural acceleration, seat belt force distribution, and dummy thoracic acceleration.

This integrated approach enables the synchronized characterization of local vehicle structural response, restraint loading, and occupant biomechanical response within a common temporal reference.

### 2.2. Anthropomorphic Test Devices and Occupant Configuration

Each vehicle was equipped with two Hybrid III 50th percentile male ATDs, as specified in [27], installed in the front seating positions. The driver position was identified as ISO-MME position 11, and the front passenger position as ISO-MME position 13. The ISO-MME position codes are used throughout the Section 3 to identify the ATD associated with each reported measurement trace.

**Figure 2:**
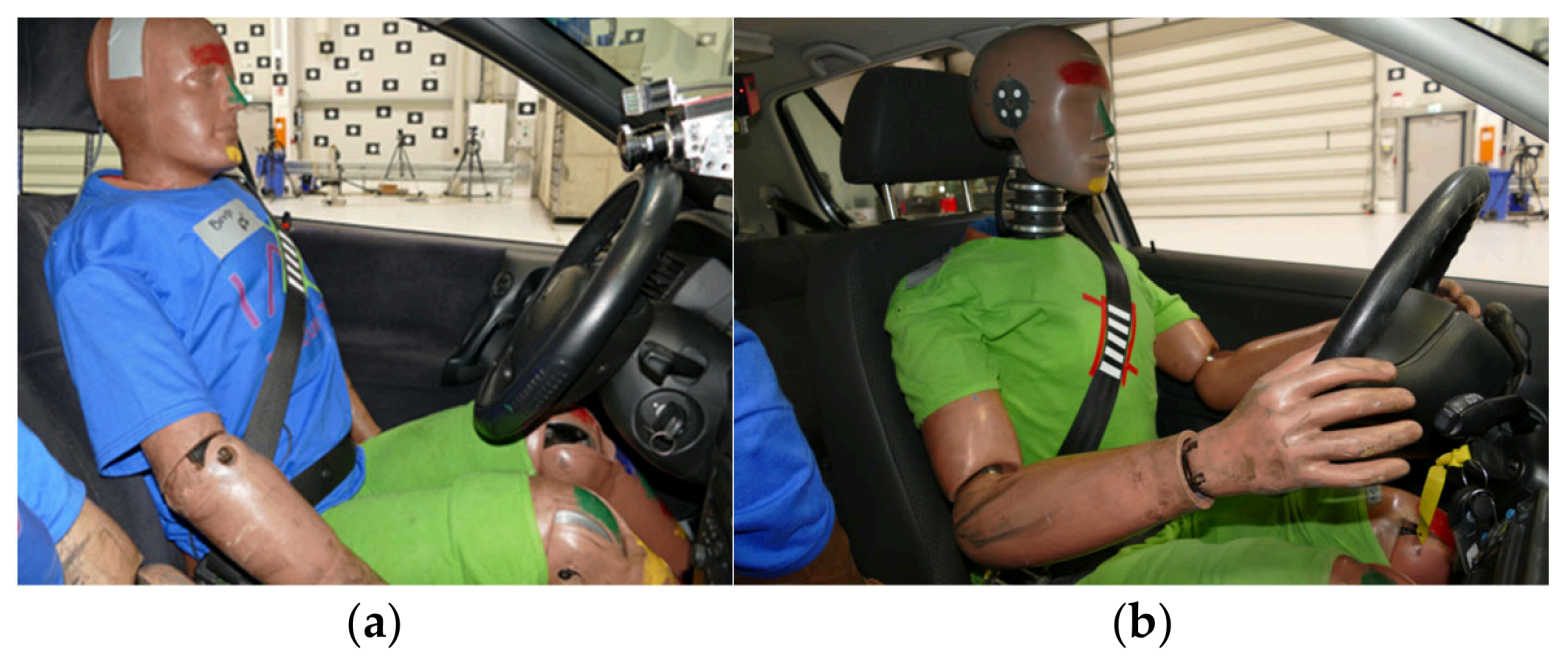
Dummy Configuration Positioning for front-rear test: (**a**) driver positioning—target vehicle; (**b**) driver positioning—striking vehicle.

For the front-to-rear impact test, the Hybrid III 50th percentile male ATDs were positioned in the driver and front-passenger seats in the standard in-position configuration (Figure 2a,b): pelvis properly aligned with the seat cushion, torso in contact with the seatback, and head oriented forward. The seating and restraint configuration followed the standard positioning procedure, with the ATDs positioned using the corresponding SAE H-point reference and restrained using the production three-point seat belt system. No intentional modification of the occupant posture or additional belt slack was introduced.

For the full-frontal rigid-barrier test, the initial seat configuration was established according to the FMVSS 208 positioning procedure for the belted Hybrid III 50th percentile male ATD. The seat was positioned at the mid fore–aft position, with the seat height set to the lowest position available at this longitudinal setting. The seat-back was adjusted to the manufacturer’s nominal design riding position for a 50th percentile male occupant; the measured seat-back inclination was approximately 20° rearward from the vertical. The lumbar support was set to its lowest, retracted, or deflated position, as applicable, and any additional adjustable seat supports were placed in their lowest or most open position. The head restraint was set to the top position, and the upper seat belt anchorage height adjustment (D-ring) was set to the upper position, in accordance with the FMVSS 208 positioning procedure.

**Figure 3:**
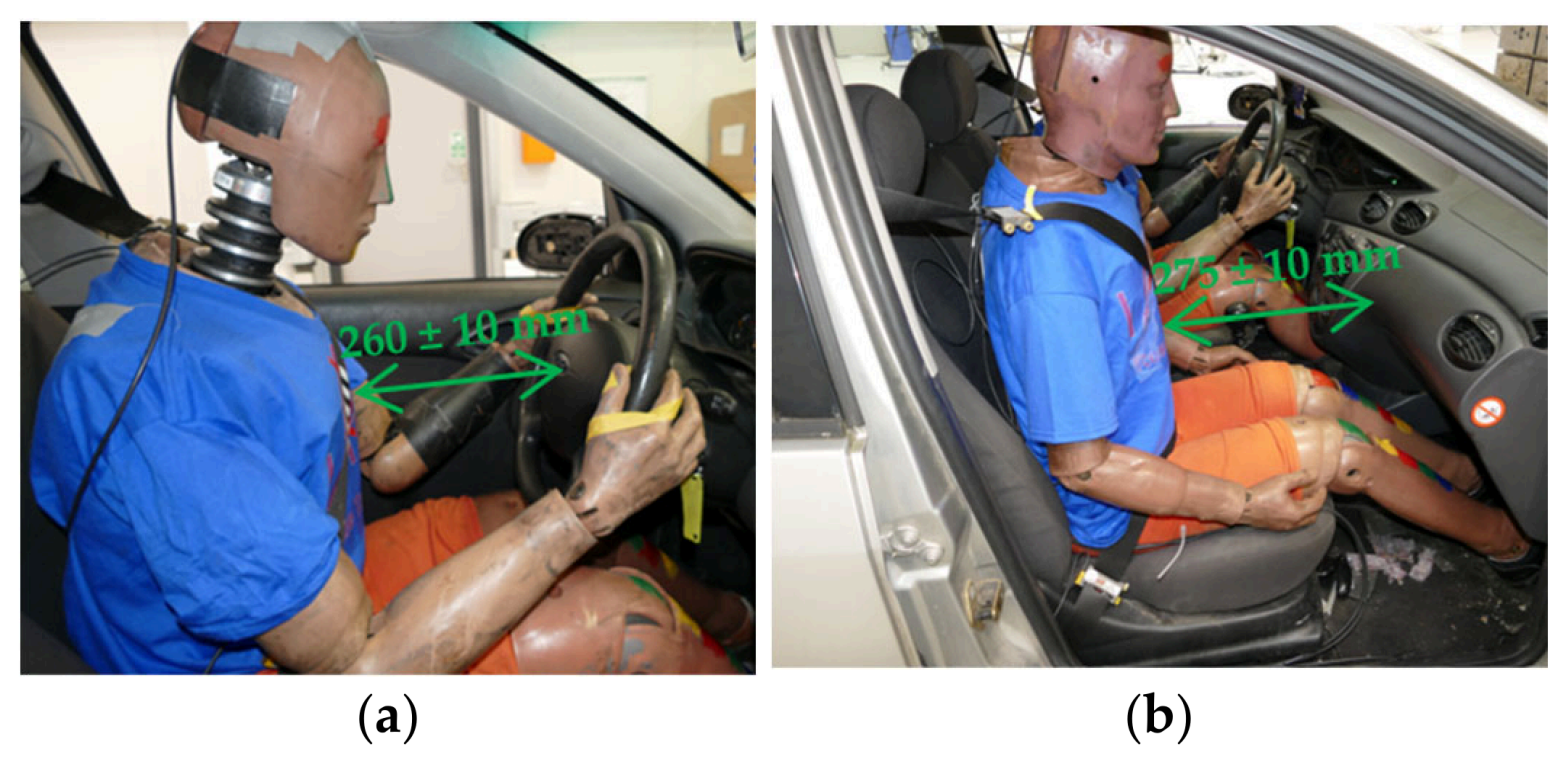
Forward-leaning OOP configuration: (**a**) driver positioning; (**b**) passenger positioning. The indicated horizontal distances were retrospectively estimated from the pre-test setup photographs using the steering-wheel diameter as the image-scaling reference.

The ATD pelvis was positioned using the corresponding SAE H-point reference. The forward-leaning OOP configuration (Figure 3a,b) was intentionally introduced to represent an occupant posture that may occur following pre-impact activation of an active safety system, such as emergency braking. For this purpose, the pelvis orientation was intentionally set outside the standard positioning range, with a measured pelvis angle of approximately 17°, compared with the reference value of 22.5° ± 2.5°. To further characterize the initial forward-leaning posture, approximate horizontal distances were retrospectively estimated from the pre-test setup photographs using a common image scale derived from the steering-wheel diameter. For the driver, the horizontal distance from the steering-wheel center to the front surface of the thorax was estimated as 260 ± 10 mm. For the front passenger, the minimum horizontal distance from the front surface of the thorax to the closest protruding point of the instrument panel was estimated as 275 ± 10 mm. These image-derived dimensions are provided as approximate geometric descriptors of the initial OOP posture and were not used as positioning criteria during the original test setup.

The production seat belt routing and anchorage geometry were maintained, and no additional belt slack was intentionally introduced. The vehicle itself was not actively braked during the crash test.

The primary biomechanical parameter analyzed in this study was chest acceleration, measured using triaxial accelerometers integrated within the thoracic cavity of each ATD. Head acceleration was also recorded using the integrated triaxial head accelerometer assembly and was used for the calculation of HIC15 reported in Appendix B. Other ATD injury measures, including chest deflection and lower-extremity injury metrics, were outside the scope of the present analysis and are therefore not evaluated in this study.

### 2.3. Vehicle Instrumentation

**Figure 4:**
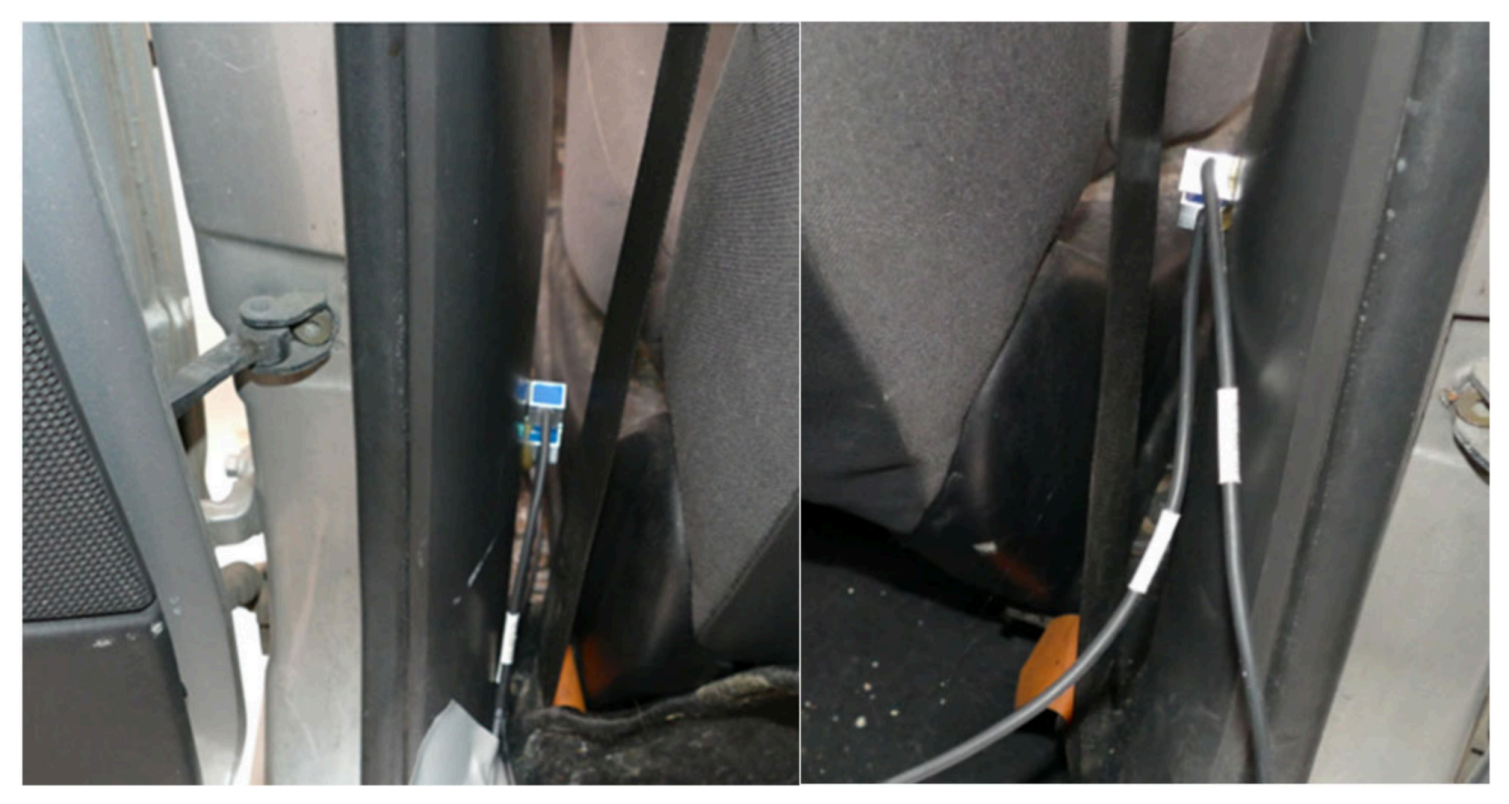
Vehicle Instrumentation: B-pillar left and right, triaxial accelerometers.

To capture vehicle-level dynamics, multiple triaxial accelerometers were installed on each test vehicle. For the present analysis, only the sensors mounted at the left and right B-pillars (Figure 4) were considered, providing measurements of the local structural acceleration response at the occupant-compartment boundary.

**Figure 5:**
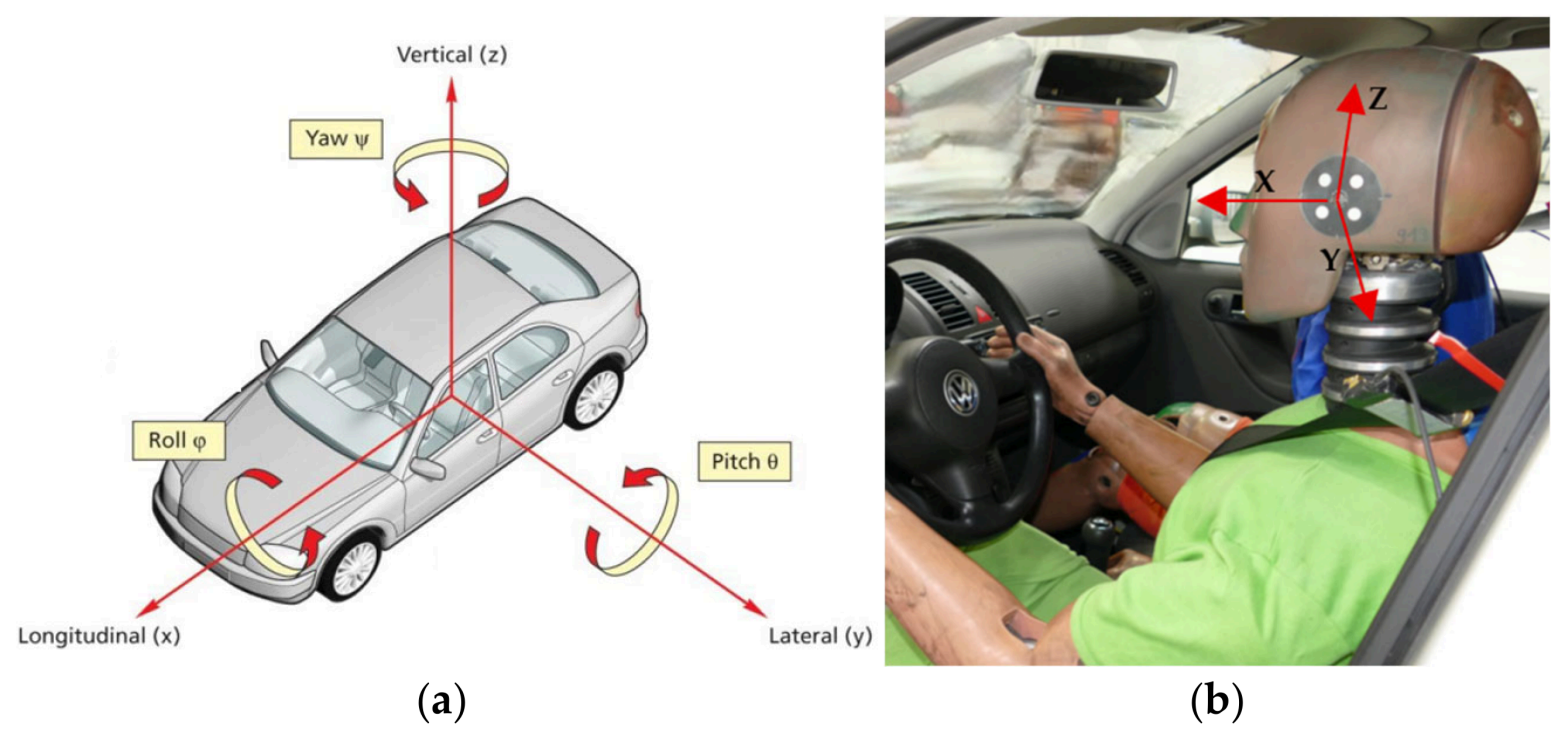
The fixed coordinate system according to ISO 8855: (**a**) Vehicle; (**b**) ATD.

The vehicle and dummy-fixed coordinate system were used according to the convention specified in ISO 8855 [28] (Figure 5a,b), with the longitudinal axis (X-direction) aligned with the direction of travel. Although three-axis data were recorded, the present analysis focuses on longitudinal acceleration, as it represents the dominant component in both frontal and rear-end impact scenarios.

The use of a consistent sensor location and orientation across all tests enables comparison of the local B-pillar acceleration responses.

### 2.4. Seat Belt Force Measurement

**Figure 6:**
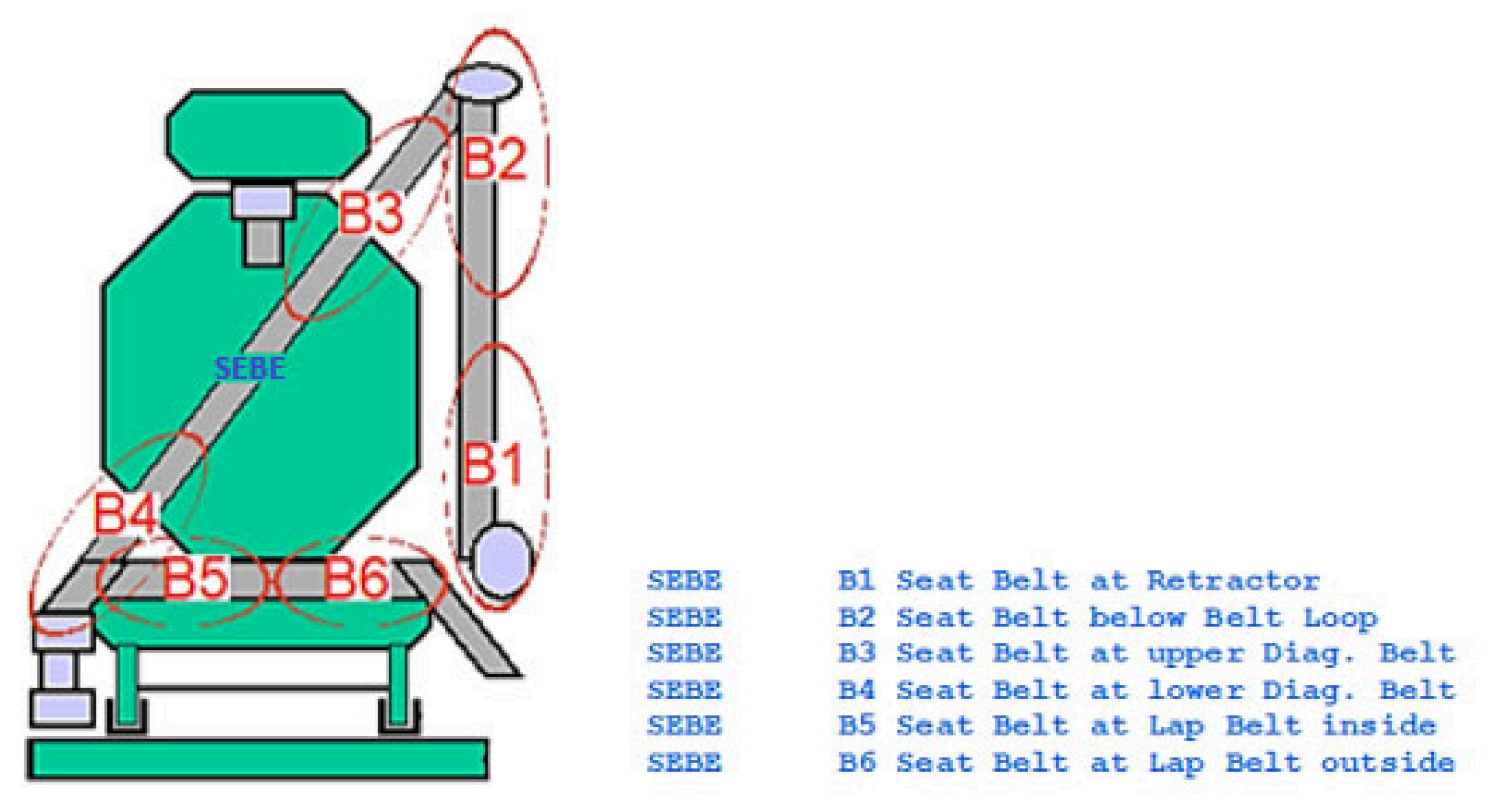
Seat belt force measurement—position and ISO-Codes [29].

To quantify restraint-system loading, force transducers were integrated into the three-point seat belt system at predefined locations (Figure 6).

These measurement points were selected according to ISO 13499 [29] to characterize force transmission along key belt segments during impact. The recorded values represent local webbing tensions at the respective measurement positions rather than the total belt force.

According to ISO 13499, the instrumented seat belt segments are identified as B3 (upper diagonal belt), B4 (lower diagonal belt), B5 (inner lap belt), and B6 (outer lap belt), and these designations are used throughout the remainder of this paper. The number of instrumented belt locations differed between the investigated configurations according to the expected restraint loading and the specific measurement objectives of each test. For the rear-end target vehicle, relatively low belt loading was anticipated because the occupant initially loads the seatback; therefore, one diagonal-belt location (B3) and one lap-belt location (B5) were instrumented. For the striking vehicle, greater upper-body restraint loading was anticipated, and two diagonal-belt locations (B3 and B4) and one lap-belt location (B6) were instrumented. For the frontal OOP configuration, the non-standard forward-leaning posture made the distribution of belt loading across the restraint system of particular interest; therefore, the complete B3–B6 set was instrumented. Accordingly, comparisons between configurations are based on the individual local webbing loads and not on a common total belt-force quantity.

**Figure 7:**
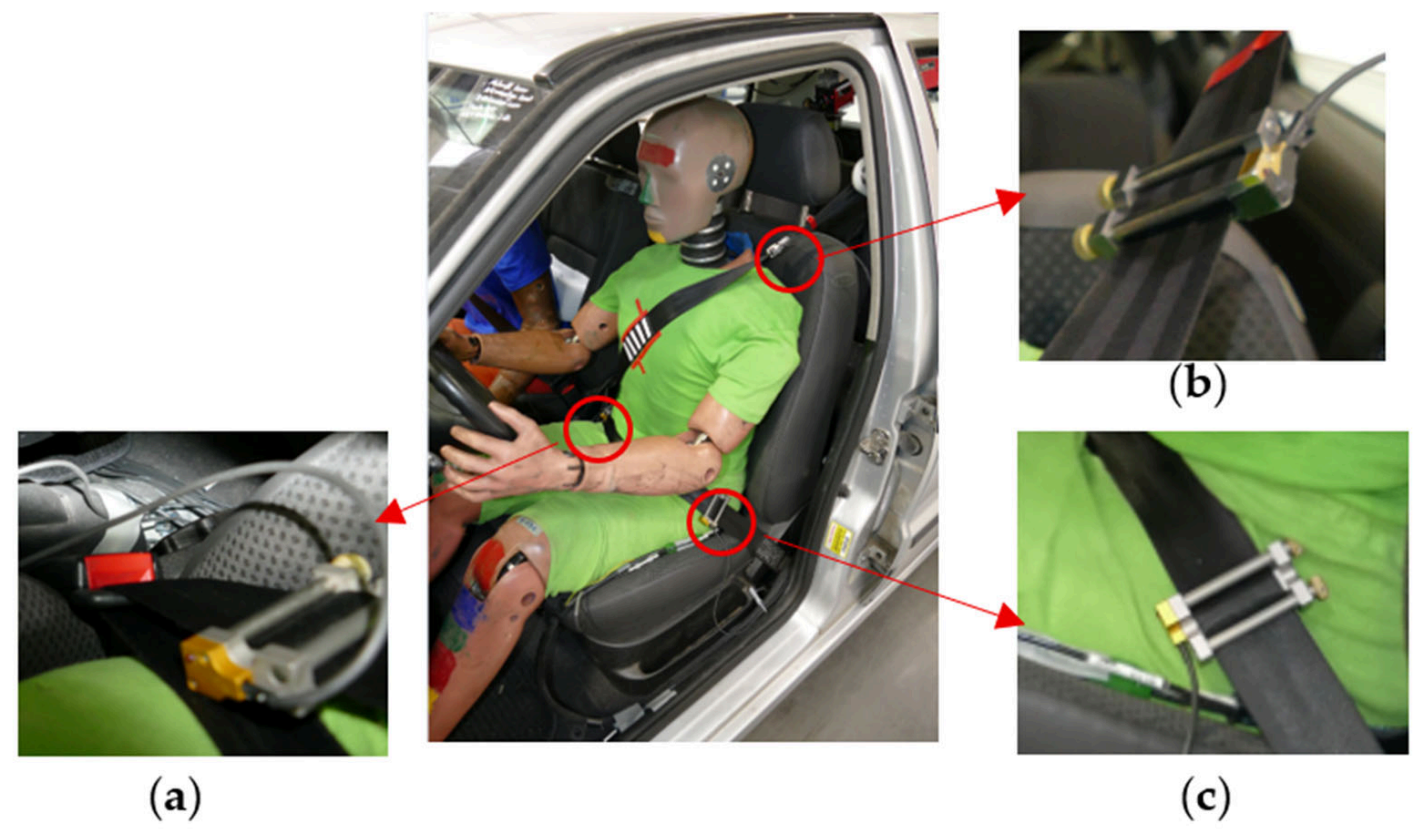
Seat belt force transducers: (**a**) B4—lower diagonal belt; (**b**) B3—upper diagonal belt; (**c**) B6—lap belt outside.

All belt force signals (Figure 7) were recorded simultaneously with vehicle and ATD acceleration data.

The tested vehicles were production-series vehicles equipped with their original restraint systems. Pretensioner and airbag activation were controlled automatically by the production restraint control unit during the crash events. In the rear-end target vehicle, the frontal airbags did not deploy, whereas the seat belt pretensioners were activated at both front seating positions. In the rear-end striking vehicle, both frontal airbags and the seat belt pretensioners were activated. In the frontal OOP configuration, both frontal airbags and the seat belt pretensioners were also activated. The present study did not investigate or characterize manufacturer-specific parameters such as pretensioner firing thresholds and timing, load-limiter characteristics and limit levels, retractor locking strategies, or airbag deployment timing. Accordingly, the measured belt-force signals are interpreted as the local webbing loads recorded at the defined measurement positions rather than as a characterization of the internal control strategy or load-limiting performance of the production restraint system.

### 2.5. Data Acquisition and Signal Processing

**Figure 8:**
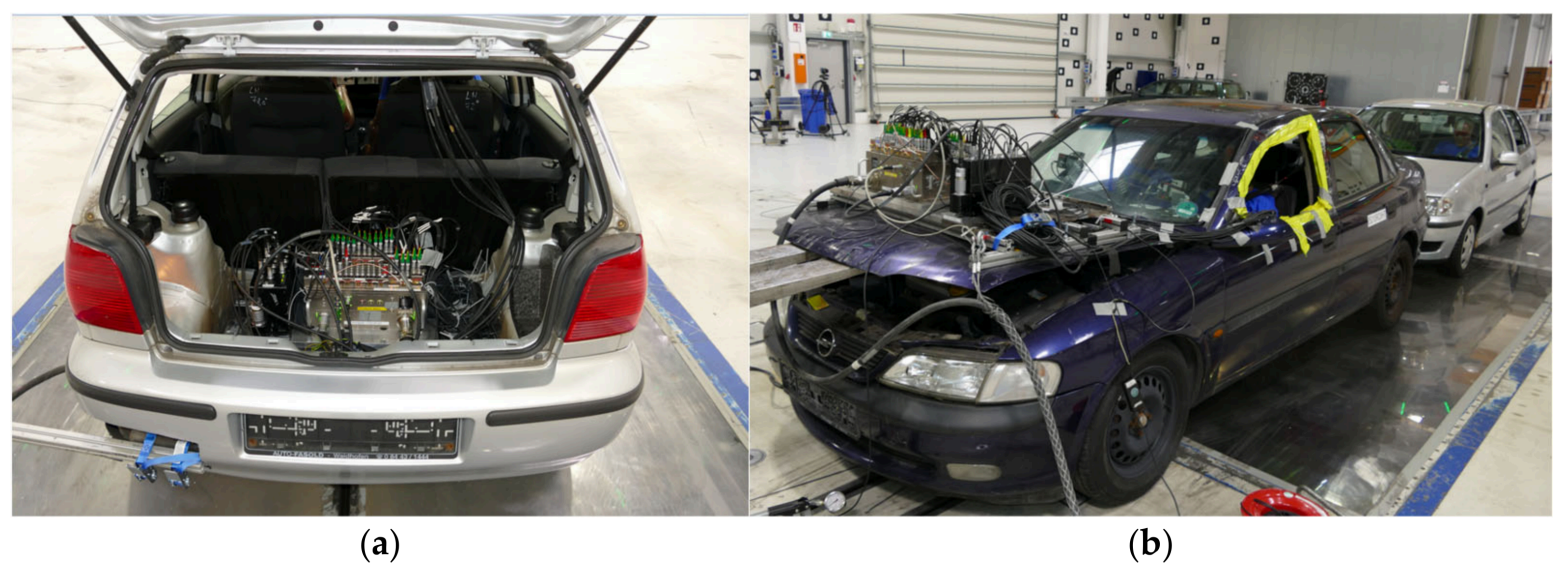
Data Acquisition System for front-rear test: (**a**) striking vehicle; (**b**) target vehicle.

Given the two distinct impact configurations described above, a dedicated data acquisition strategy was implemented for each test setup to ensure measurement consistency and signal integrity. The sensors were connected to the corresponding data-acquisition (DAQ) units used for each test configuration (Figure 8).

In the front-to-rear configuration, the DAQ unit of the striking vehicle was installed in the luggage compartment to ensure protection during the collision. The stationary target vehicle was equipped with a separate acquisition unit mounted on the front hood area. In the full-frontal rigid-barrier configuration, the DAQ unit was installed in the luggage compartment of the test vehicle.

Signals from the B-pillar accelerometers, ATD thoracic accelerometers, and seat belt force transducers (B3–B6) were recorded simultaneously using synchronized multi-channel acquisition systems. Vehicle acceleration was measured using triaxial accelerometers (Advanced International Sensors) mounted on the B-pillars. Thoracic acceleration was acquired using the standard triaxial accelerometer integrated into the Hybrid III 50th percentile male ATD. Seat belt forces were measured using force transducers (ME-Messsysteme GmbH). The main technical specifications of the instrumentation associated with the measurements analyzed in this study are summarized in Appendix A.

Signal acquisition and processing were performed in accordance with established impact-test measurement practices and the recommendations of SAE J211/1 and ISO 6487 [30,31]. All channels were sampled at 20 kHz. Signal processing was performed using ISO Verter V1.7.4 software. B-pillar acceleration and seat belt force signals were filtered using Channel Frequency Class (CFC) 60, thoracic acceleration signals using CFC 180, and head acceleration signals used for HIC15 calculation using CFC 1000. The CFC implementation in ISO Verter uses a fourth-order phaseless (acausal) Butterworth filter; therefore, the filtering procedure does not introduce a phase shift between the processed measurement channels.

The experimental tests were conducted within an accredited vehicle-safety testing laboratory operating in accordance with DIN EN ISO/IEC 17025:2018. Measurement equipment, data-acquisition systems, calibration procedures, and signal processing were managed within the laboratory quality system and in accordance with the applicable crash-test measurement standards, including SAE J211/1 and ISO 6487 [30,31]. The present study focuses on the synchronized multi-sensor methodology and does not include a channel-specific measurement uncertainty budget.

The reference time (t_0_) was defined by the physical impact trigger. For the front-to-rear collision, a physical contact trigger mounted on the rear of the target vehicle generated the t_0_ signal at first contact between the vehicles. The signal was transmitted simultaneously to the acquisition units installed in the striking and target vehicles, thereby establishing a common temporal reference for both measurement systems. The temporal alignment was independently verified using the synchronized high-speed video recording, in which the frame corresponding to t = 0 ms showed the first physical contact between the two vehicles. For the full-frontal rigid-barrier test, a physical contact trigger mounted on the rigid barrier defined t_0_ at the first contact of the vehicle with the barrier.

Acceleration measurements are expressed in units of gravitational acceleration (g), consistent with the conventions adopted in automotive crash testing standards and regulations.

### 2.6. Data Analysis Strategy

**Figure 9:**
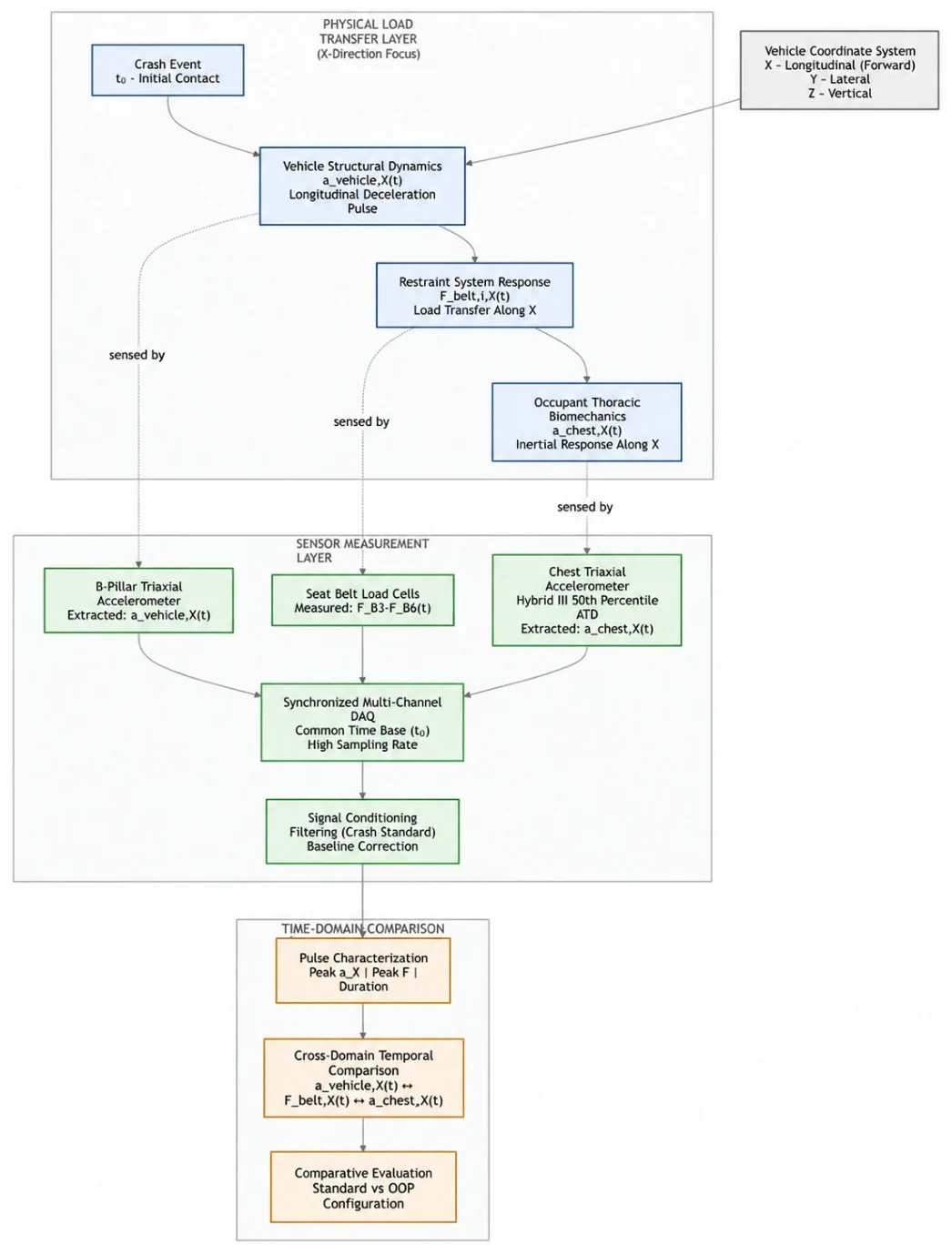
System-Level Framework for Multi-Sensor Assessment of Vehicle and Occupant Dynamics.

The analysis focused on the synchronized characterization of local vehicle structural response, restraint-system loading, and occupant thoracic response within each investigated crash configuration (Figure 9). The evaluated parameters included longitudinal B-pillar acceleration (X-direction), ATD thoracic acceleration, and local seat belt webbing tensions measured at the instrumented B3–B6 positions. Peak values and corresponding peak times were extracted from the processed time-history signals.

Comparisons between the investigated configurations were performed descriptively, with the observed differences interpreted as configuration-specific experimental observations rather than as isolated effects of occupant posture or other individual test parameters.

By integrating vehicle structural, restraint-system, and occupant biomechanical measurements within a synchronized framework, the methodology enables a consistent multi-domain characterization of occupant–vehicle interaction during crash events.

Acceleration time histories are presented with their original sign according to the coordinate convention defined in Section 2.3. For summary comparison in Table 2, peak acceleration values are reported as absolute magnitudes, whereas seat belt forces are reported as positive tensile loads.

## 3. Results

This section presents the results obtained from crash tests conducted under two impact configurations: a rear-end collision between two vehicles and a frontal impact against a rigid barrier. For all tests, two Hybrid III 50th percentile male ATDs were placed on the front seats. In the rear-end scenario, the dummies were positioned in the standard seating position, while in the frontal barrier test, they were placed out-of-position (OOP), leaning forward relative to the seatback.

The analysis focuses on vehicle structural response, occupant thoracic response, and restraint-system loading, evaluated through B-pillar acceleration, thoracic acceleration, and local seat belt webbing tensions. Results are reported separately for each vehicle, including peak values and representative high-speed camera frames. The following sections present the detailed structural and occupant responses for each tested vehicle, organized by impact scenario and measurement type.

### 3.1. Rear-End Impact Configuration

#### 3.1.1. Target Vehicle (Rear-Impacted Vehicle)

- Vehicle Structural Response

**Figure 10:**
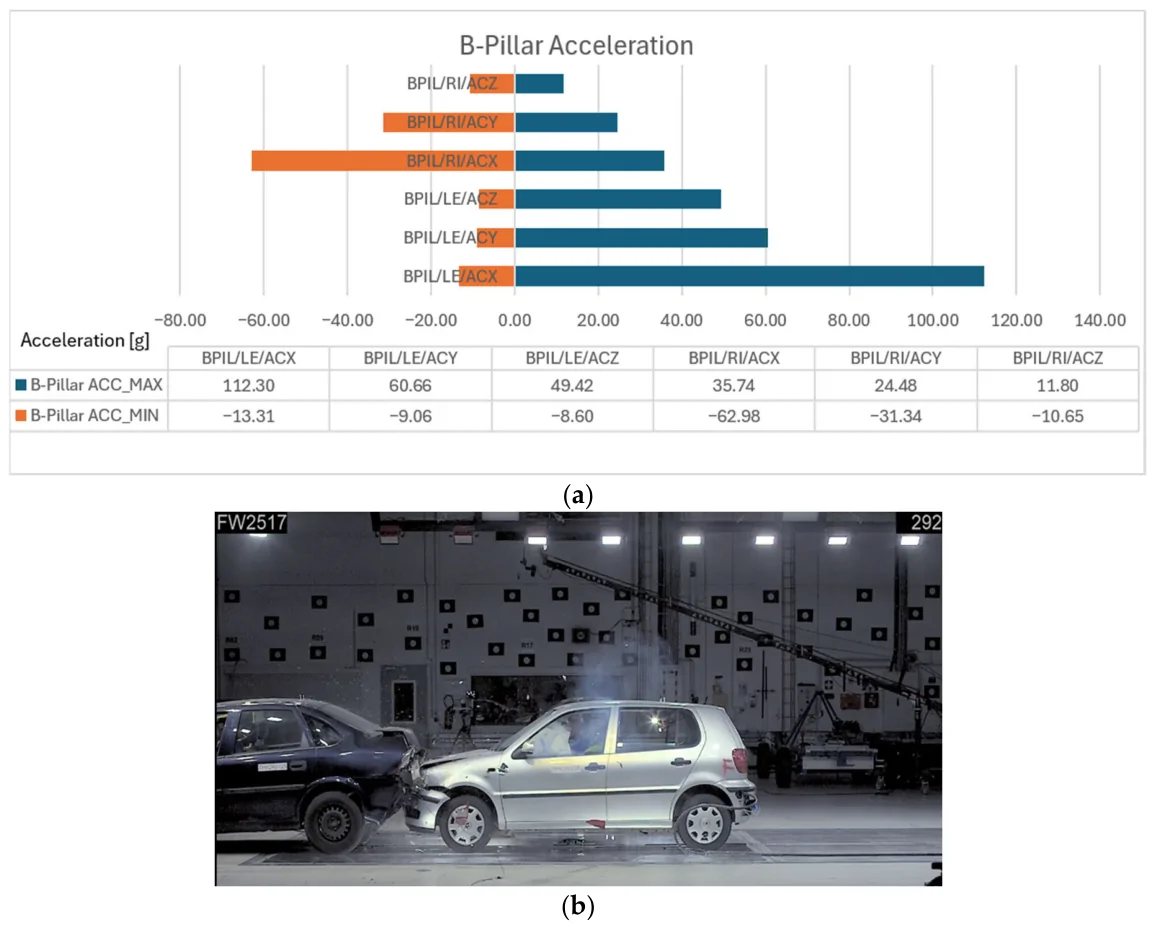
Target vehicle structural responses in rear-end impact: (**a**) B-pillar acceleration measured in the rear-impacted vehicle; (**b**) high-speed camera frames corresponding to the moment of peak acceleration.

The B-pillar acceleration recorded for the rear-impacted vehicle is presented in Figure 10a. Triaxial measurements were obtained, with the longitudinal (*X*-axis) component representing the dominant direction of the measured response. A peak local longitudinal B-pillar acceleration magnitude of 112.30 g was recorded at 291.85 ms. This value represents the local structural response at the B-pillar measurement location and should not be interpreted as the translational acceleration of the vehicle as a whole.

The corresponding high-speed camera frame at the time of peak B-pillar acceleration is shown in Figure 10b. At this instant, the two vehicles were still in physical contact, with the rear-impacted vehicle continuing to be pushed forward by the striking vehicle during the late stage of the vehicle-to-vehicle interaction. The peak at 291.85 ms therefore occurred during the prolonged contact phase associated with the specific underride-type engagement, rather than after vehicle separation.

- Occupant Response

**Figure 11:**
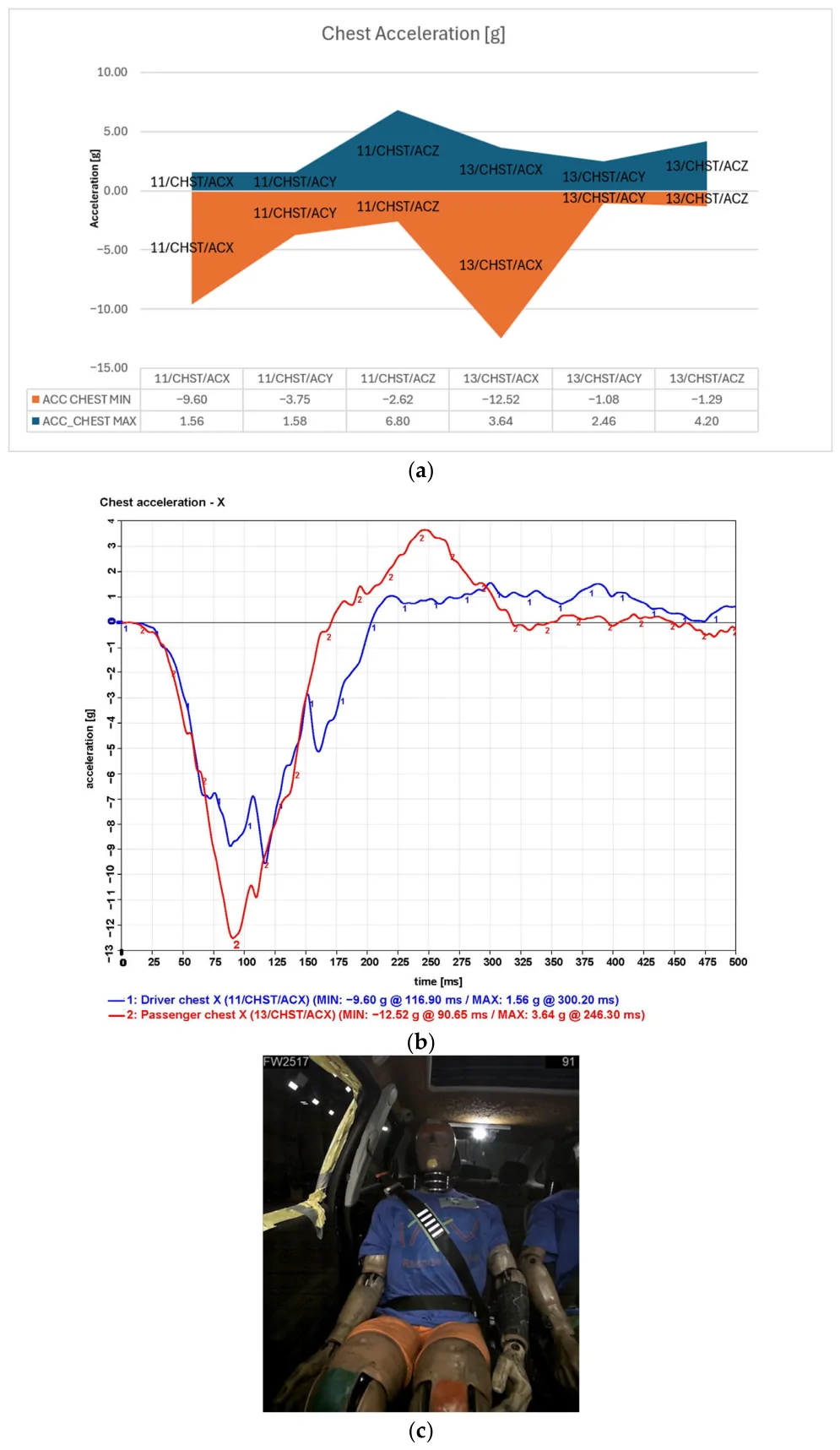
Thoracic acceleration measured at the ATD chest during the rear-end collision—Target Vehicle: (**a**) maximum and minimum accelerations for each ATD across the *X*-, *Y*-, and *Z*-axes; (**b**) longitudinal (*X*-axis) thoracic acceleration time history; (**c**) high-speed camera frame corresponding to the moment of peak acceleration.

The thoracic acceleration measured at the ATD chest level is illustrated in Figure 11a,b. Following impact, the target vehicle is accelerated forward while the ATDs initially exhibit relative rearward motion with respect to the vehicle interior due to inertia. The recorded thoracic acceleration subsequently increases as the occupant response develops during the event. The high-speed camera frame corresponding to the peak thoracic acceleration is presented in Figure 11c and illustrates the ATD posture at that instant.

- Seat Belt Forces in Rear-End Collision

**Figure 12:**
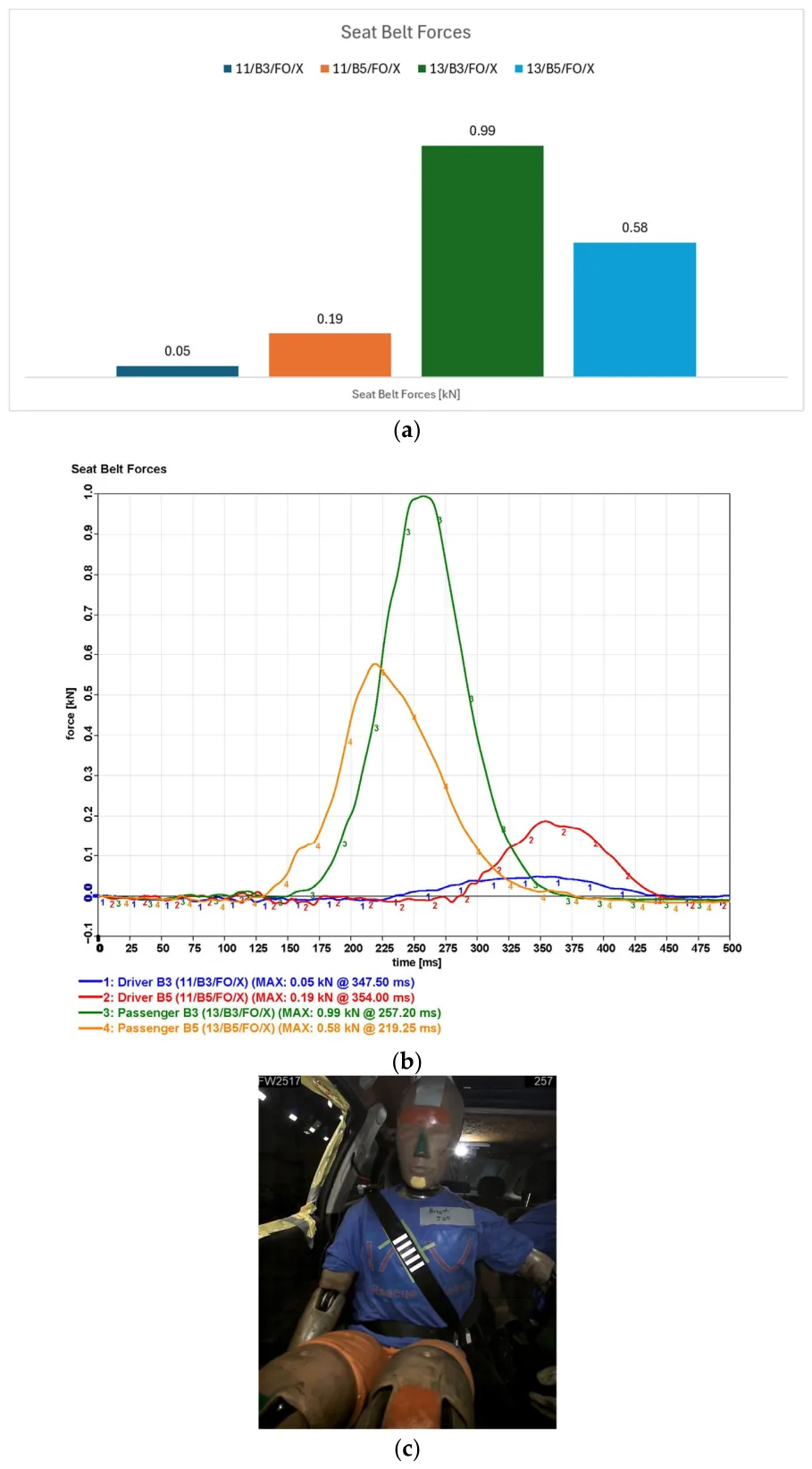
Seat belt forces measured at channels B3 and B5 during the rear-end collision: (**a**) maximum force values; (**b**) local webbing tensions measured at the instrumented belt segments; (**c**) high-speed camera frame with the dummy position corresponding to the moment of peak force.

Seat belt force measurements obtained from the instrumented belt segments are shown in Figure 12a,b. The highest local webbing tension was recorded for the passenger at B3, reaching 0.99 kN at approximately 257 ms, while B5 exhibited a peak value of 0.58 kN.

The maxima of the belt-force signals occurred at different times from the peak local B-pillar and thoracic accelerations. In the rear-impacted vehicle, the relatively late belt-force peaks at 219.25 ms (B5) and 257.20 ms (B3) are consistent with the expected occupant kinematics of a rear impact, in which the occupant initially loads the seatback and subsequently engages the seat belt during the rebound phase. These peak times are reported as descriptive characteristics of the synchronized signals and are not interpreted as physical load-transfer delays. The high-speed camera frame corresponding to the selected peak belt-force instant is shown in Figure 12c.

#### 3.1.2. Striking Vehicle (Standard ATD Positioning)

- Vehicle Structural Response

**Figure 13:**
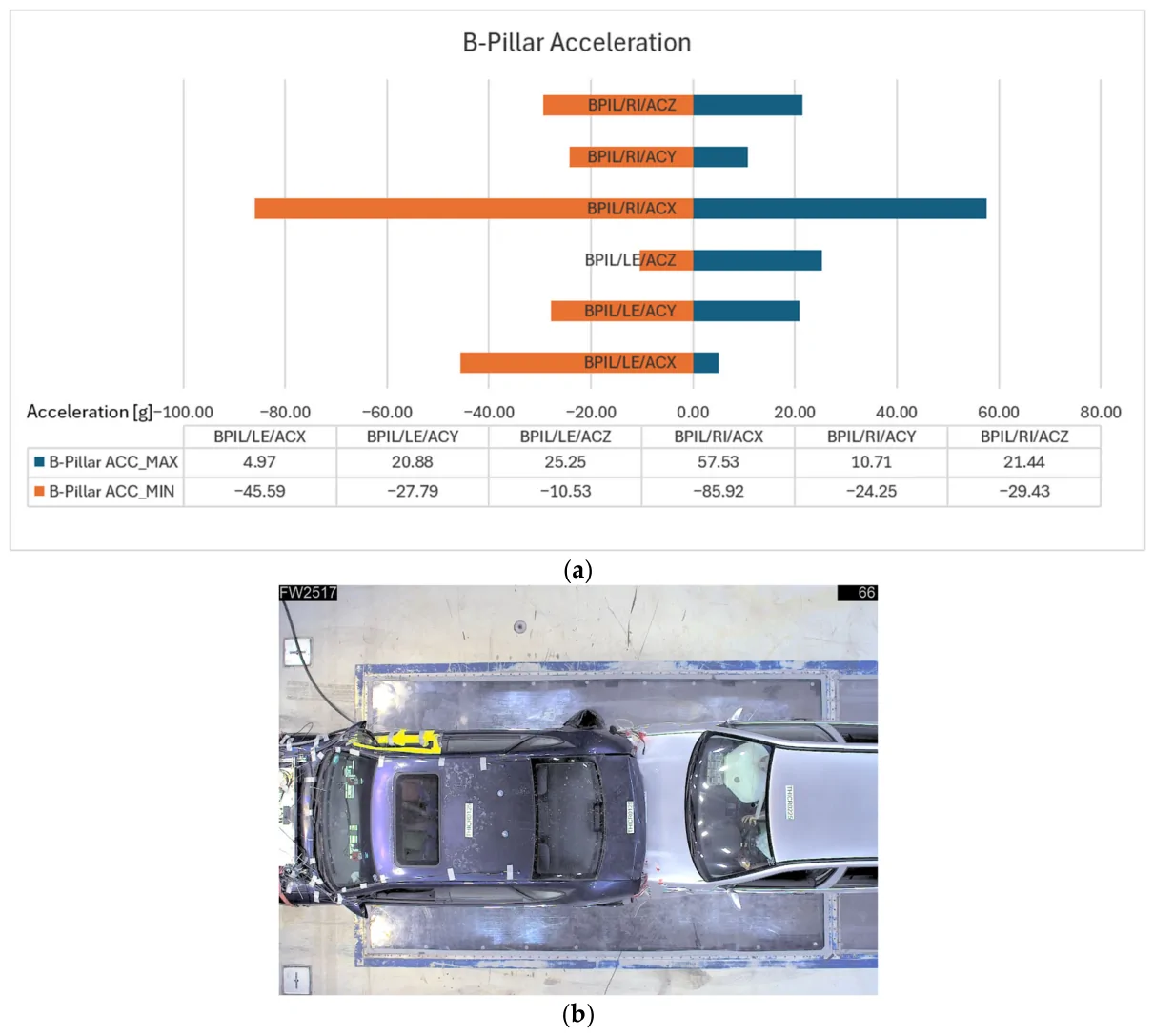
Striking vehicle structural responses in rear-end impact: (**a**) B-pillar acceleration measured in the striking vehicle; (**b**) high-speed camera frames corresponding to the moment of peak acceleration.

The B-pillar acceleration recorded for the striking vehicle is presented in Figure 13a.

Following contact with the target vehicle, the B-pillar measurement exhibited a peak local longitudinal acceleration magnitude of 85.92 g at 65.60 ms after initial contact. This value represents the local structural response at the B-pillar measurement location and should not be interpreted as the translational acceleration of the vehicle as a whole.

The corresponding high-speed camera frame is shown in Figure 13b, illustrating the structural interaction between the front structure of the striking vehicle and the rear section of the target vehicle at this instant.

- Occupant Response

**Figure 14:**
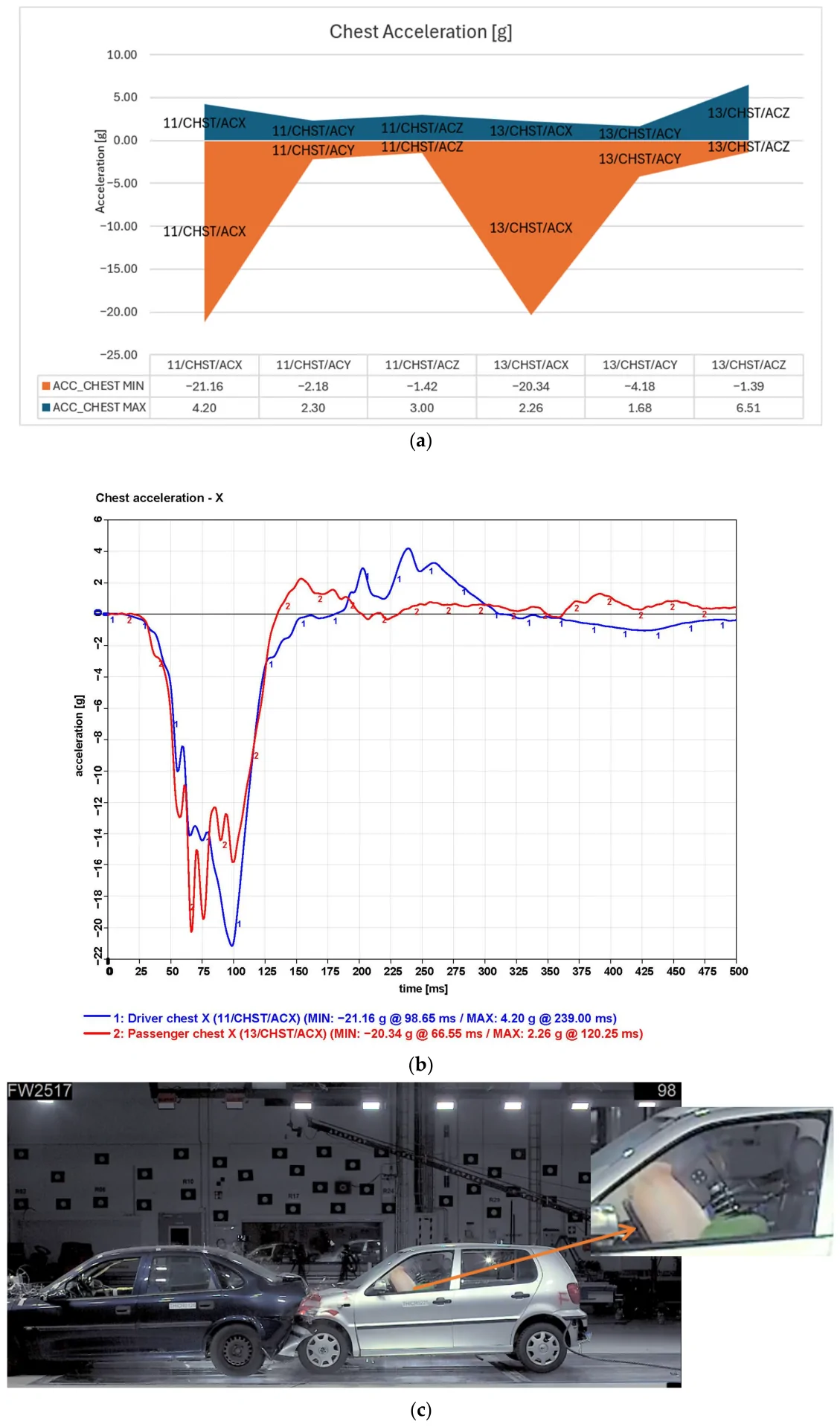
Thoracic acceleration measured at the ATD chest during the rear-end collision—Striking Vehicle: (**a**) maximum and minimum accelerations for each ATD across the *X*-, *Y*-, and *Z*-axes; (**b**) longitudinal (*X*-axis) thoracic acceleration time history; (**c**) high-speed camera frame corresponding to the moment of peak thoracic acceleration.

The thoracic acceleration recorded for the ATDs in the striking vehicle is presented in Figure 14a,b. The peak longitudinal thoracic acceleration reached 21.16 g at 98.65 ms. The corresponding high-speed camera frame is presented in Figure 14c, illustrating the forward motion of the ATD torso and its interaction with the restraint system at this instant.

- Seat Belt Forces

**Figure 15:**
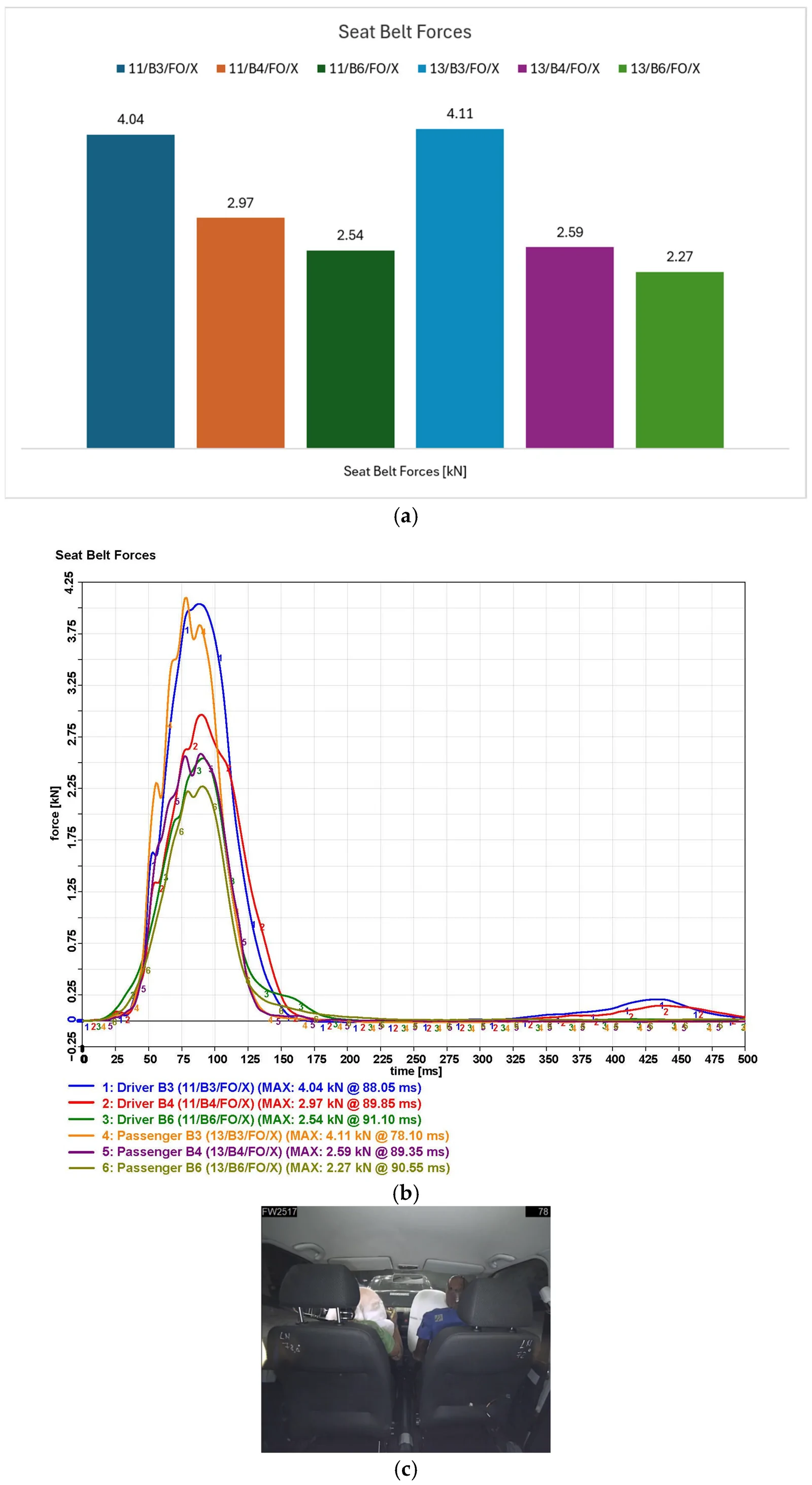
Seat belt forces measured at channels B3, B4 and B6 during the rear-end collision: (**a**) maximum force values; (**b**) local webbing tensions measured at the instrumented belt segments; (**c**) high-speed camera frame with the dummy position corresponding to the moment of peak force.

Local webbing tensions measured at the instrumented belt segments are shown in Figure 15a,b. The highest value was recorded at B3, reaching 4.11 kN, while B4 and B6 exhibited peak values of 2.97 kN and 2.54 kN, respectively.

The synchronized measurements show that the peak local B-pillar acceleration occurred first, at 65.60 ms, followed by the peak seat belt force at 78.10 ms and the peak thoracic acceleration at 98.65 ms. These values describe the relative timing of the signal maxima within the striking-vehicle configuration and should not be interpreted as direct measures of the onset or causal sequence of load transfer.

### 3.2. Full-Frontal Rigid-Barrier Impact (OOP Seating)

- Vehicle Structural Response

**Figure 16:**
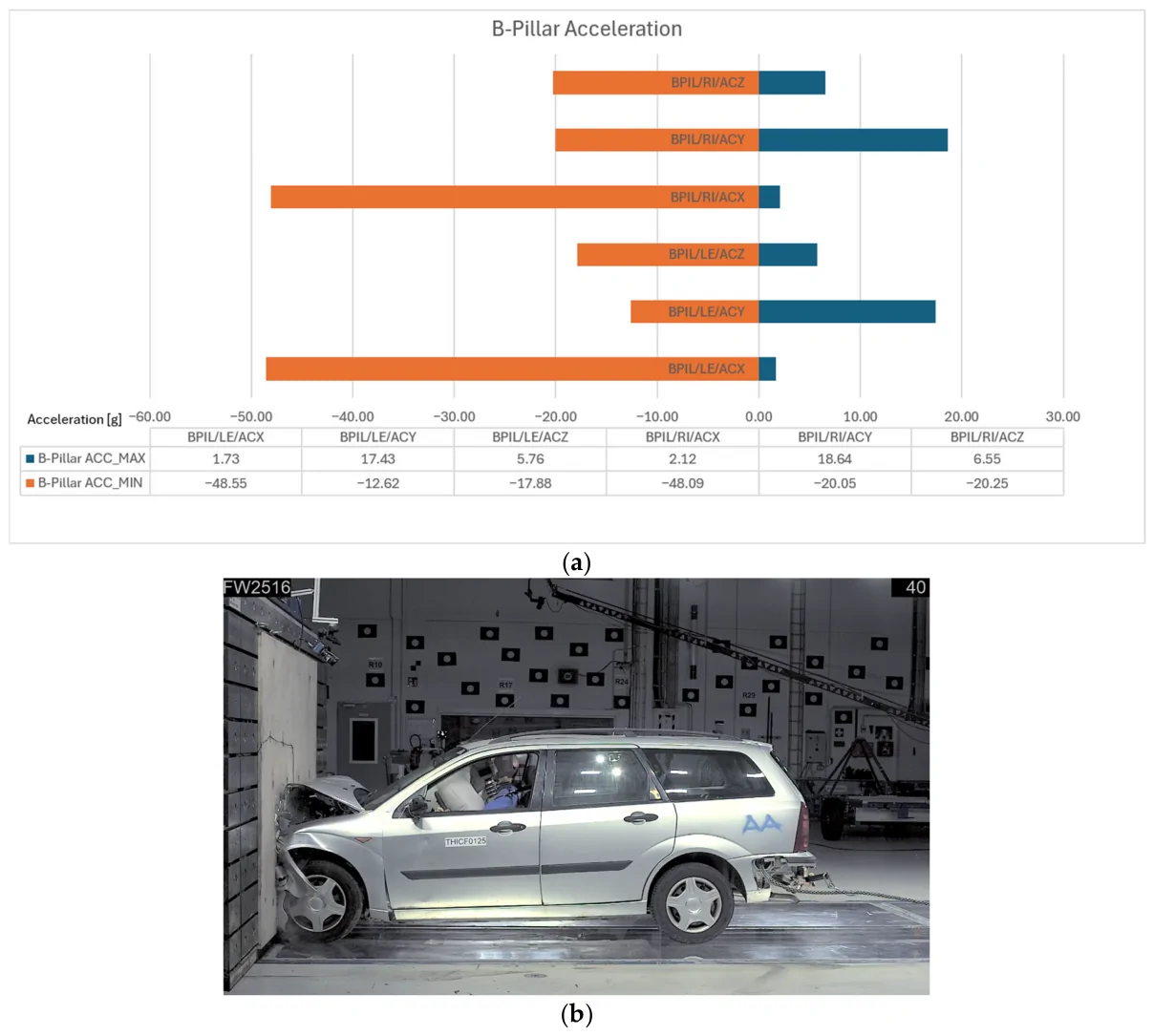
Vehicle structural response in the full-frontal rigid-barrier test: (**a**) peak B-pillar longitudinal acceleration; (**b**) high-speed camera frame corresponding to the moment of peak acceleration.

The B-pillar acceleration profile is presented in Figure 16a. Following contact with the rigid barrier, the B-pillar measurement exhibited a peak local longitudinal acceleration magnitude of 48.55 g at 39.70 ms. This value represents the local structural response at the B-pillar measurement location and should not be interpreted as the translational acceleration of the vehicle as a whole.

The corresponding high-speed camera frame is shown in Figure 16b, illustrating the deformation state of the vehicle front structure at the corresponding instant.

- Occupant Response (OOP)

**Figure 17:**
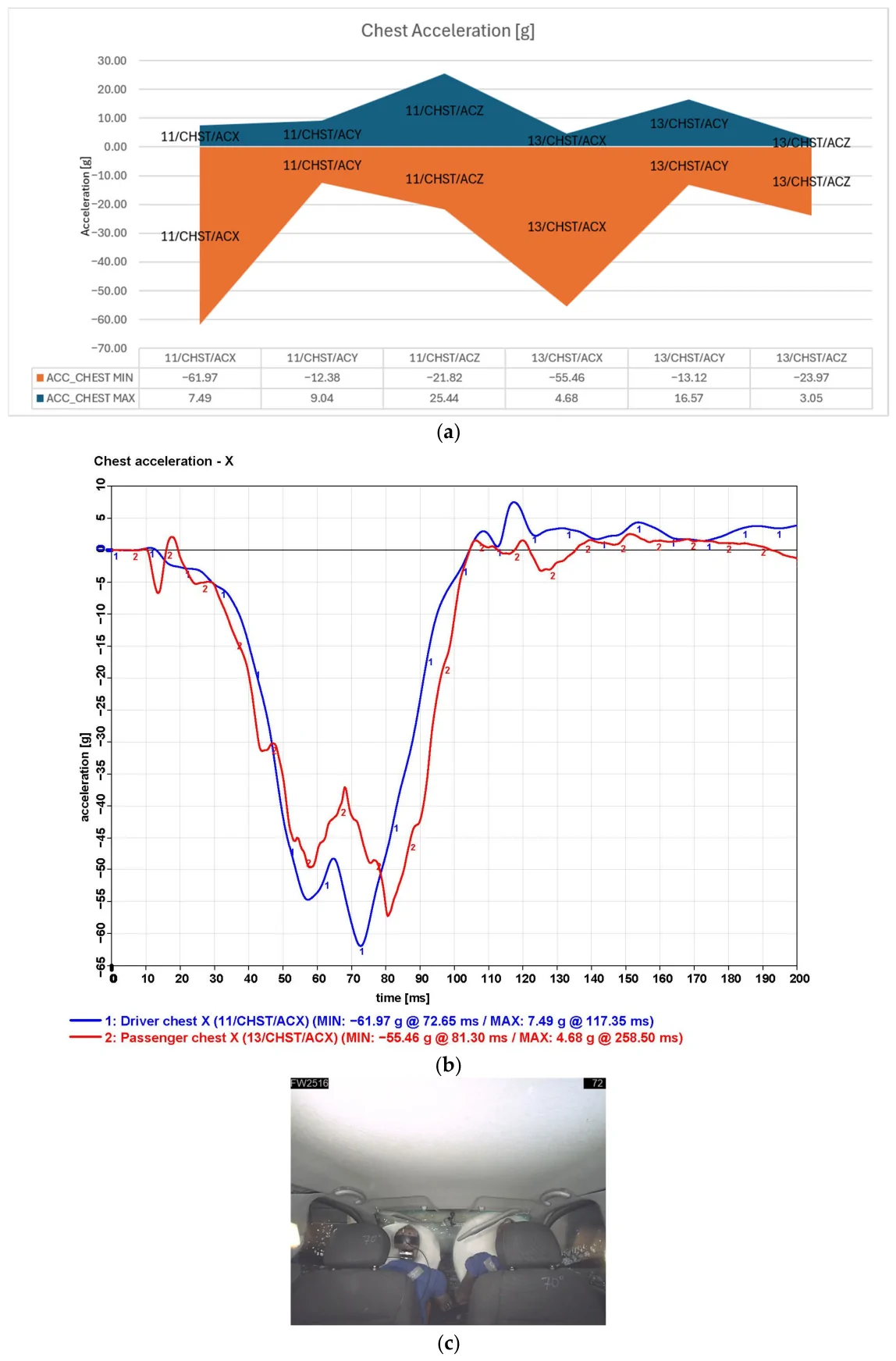
Thoracic acceleration measured at the ATD chest during the full-frontal rigid-barrier test: (**a**) maximum and minimum accelerations for each ATD across the *X*-, *Y*-, and *Z*-axes; (**b**) longitudinal (*X*-axis) thoracic acceleration time history; (**c**) high-speed camera frame corresponding to the moment of peak thoracic acceleration.

The thoracic acceleration response of the ATDs in the forward-leaning OOP configuration is presented in Figure 17a,b. The maximum recorded longitudinal thoracic acceleration was 61.97 g at 72.65 ms. The corresponding high-speed camera frame is shown in Figure 17c, illustrating the interaction between the ATD torso and the restraint system during forward motion.

- Seat Belt Forces

**Figure 18:**
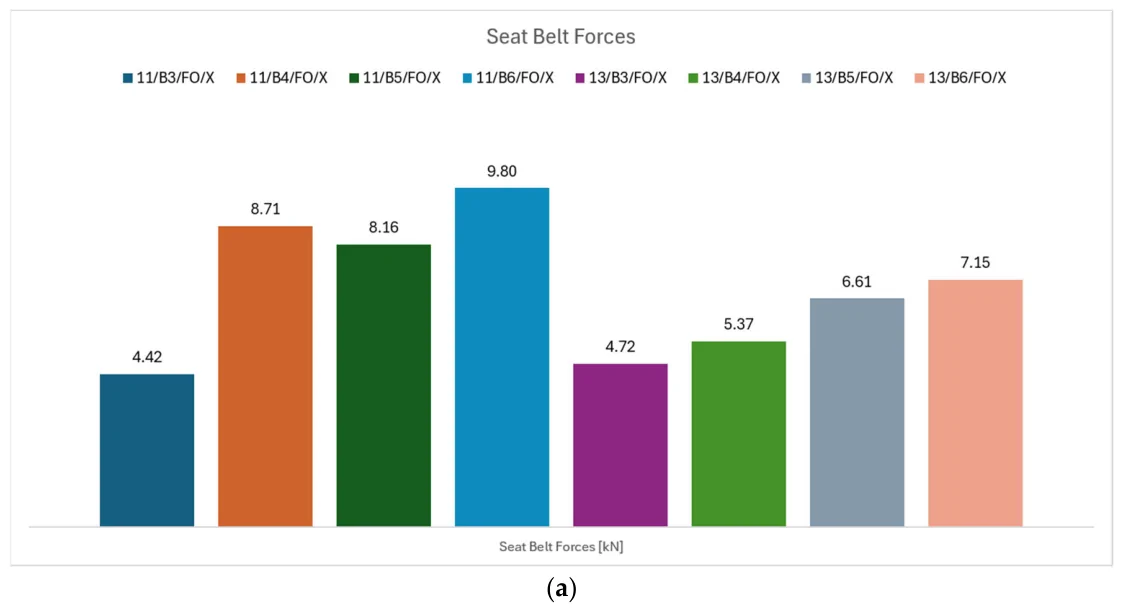

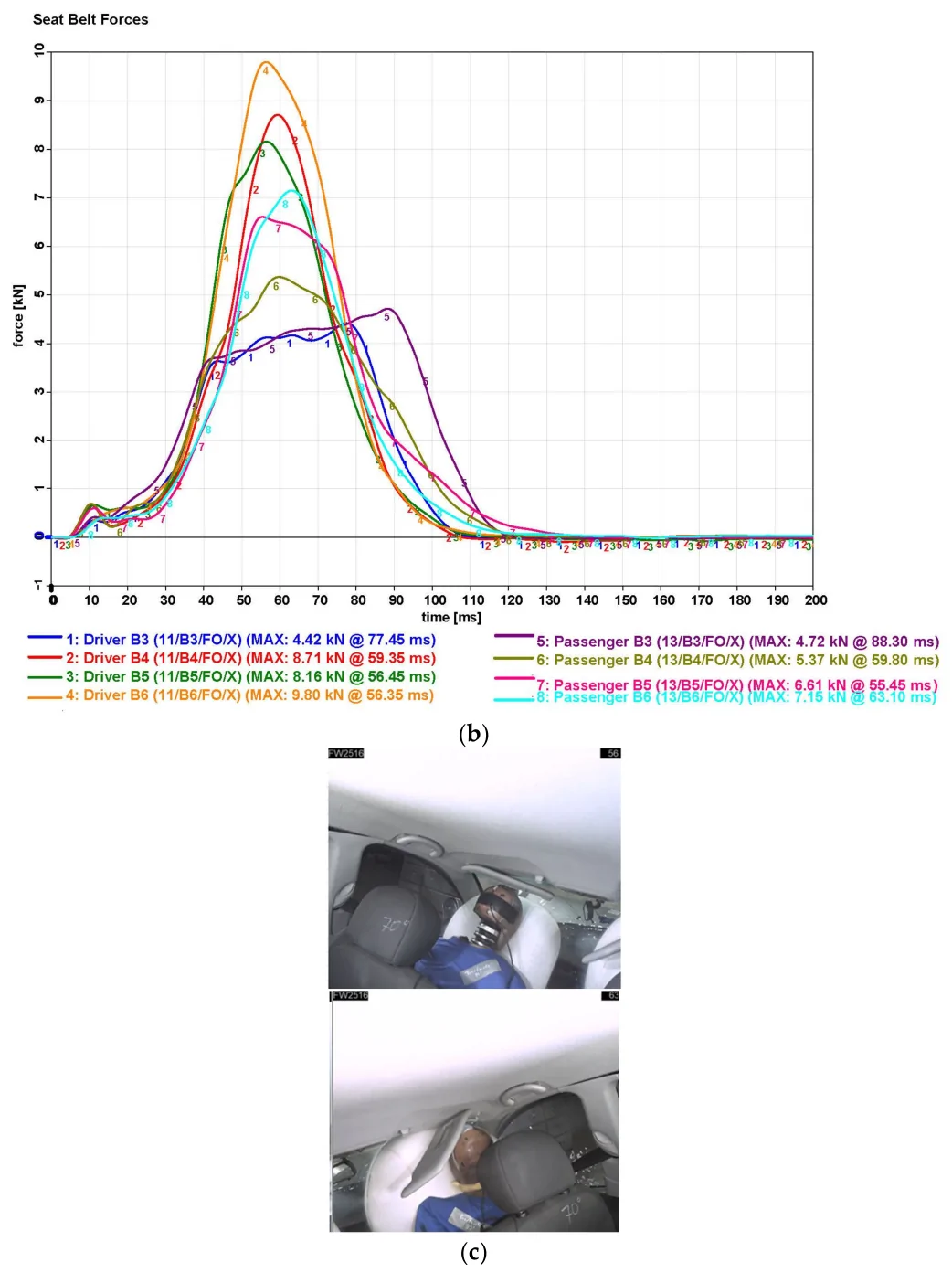
Seat belt measurements at channels B3, B4, B5, and B6 during the full-frontal rigid-barrier test: (**a**) maximum values; (**b**) local webbing tensions measured at the instrumented belt segments; (**c**) high-speed camera frame corresponding to the moment of peak local webbing tension.

Local webbing tensions measured in the frontal OOP configuration are presented in Figure 18a,b. Peak values of 4.72 kN, 8.71 kN, 8.16 kN, and 9.80 kN were recorded at B3, B4, B5, and B6, respectively. The corresponding time histories characterize the local restraint-system loading observed during the forward motion of the ATDs in this specific test configuration.

Table 2 summarizes the maximum recorded structural, occupant, and restraint responses for each investigated crash configuration. The individual ATD responses associated with ISO-MME positions 11 and 13 are presented in the preceding figures, while the table reports the highest measured value observed within each configuration. The highest local B-pillar acceleration peak was recorded in the rear-end target vehicle (112.30 g), whereas the frontal OOP configuration exhibited the highest recorded longitudinal thoracic acceleration (61.97 g) and the highest local webbing tension, reaching 9.80 kN. The B-pillar measurements represent local structural responses at the sensor mounting locations and should not be interpreted as direct measures of whole-vehicle deceleration or overall impact severity. Consequently, their peak amplitudes are not directly comparable with estimates based on vehicle center-of-gravity delta-V and overall crash-pulse duration. These observations describe configuration-specific responses and should not be interpreted as isolating the effect of occupant posture, since the investigated crash configurations differ in multiple experimental factors.

Overall, the synchronized measurements demonstrate the ability of the proposed multi-sensor framework to characterize local structural, occupant, and restraint-system responses across different crash configurations within a common experimental approach.

## 4. Multi-Sensor Response Analysis

To complement the peak-value analysis presented in the preceding section, the synchronized time-history measurements were examined to compare the temporal development of the local B-pillar acceleration, seat belt forces, and ATD thoracic acceleration within each crash configuration.

**Figure 19:**
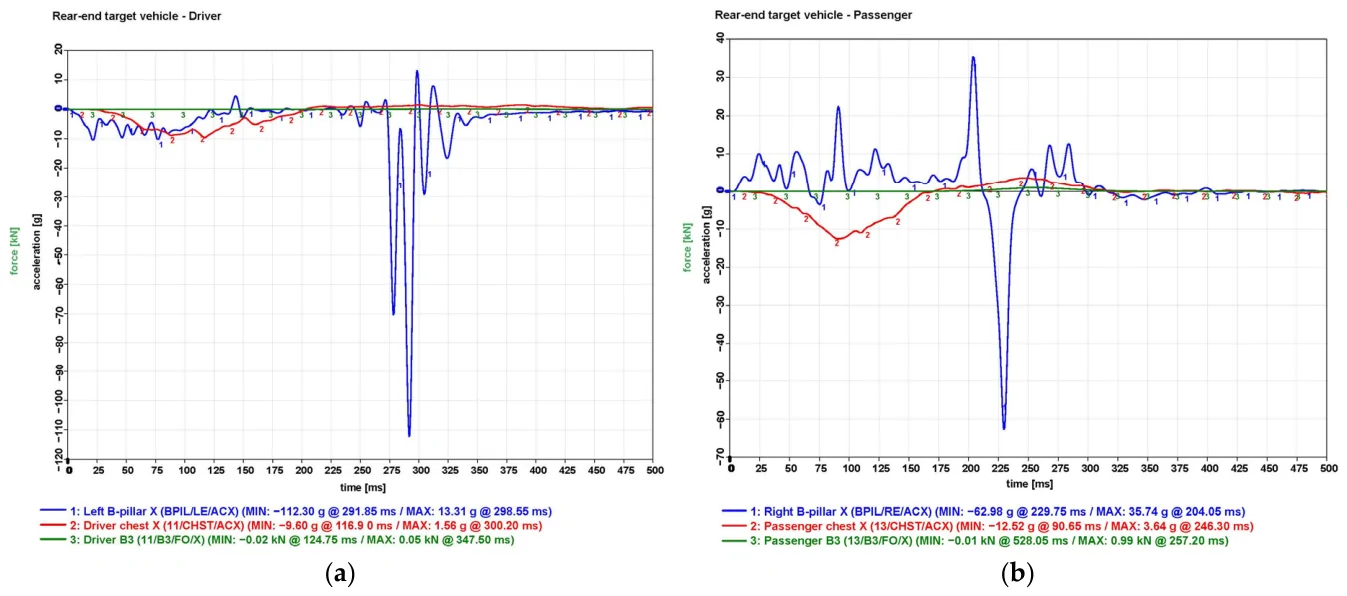
Synchronized multi-sensor responses for the rear-end target vehicle: (**a**) driver position (11); (**b**) front passenger position (13). Longitudinal B-pillar acceleration, thoracic acceleration, and local B3 seat belt webbing tension are shown relative to the common impact reference time t_0_.

**Figure 20:**
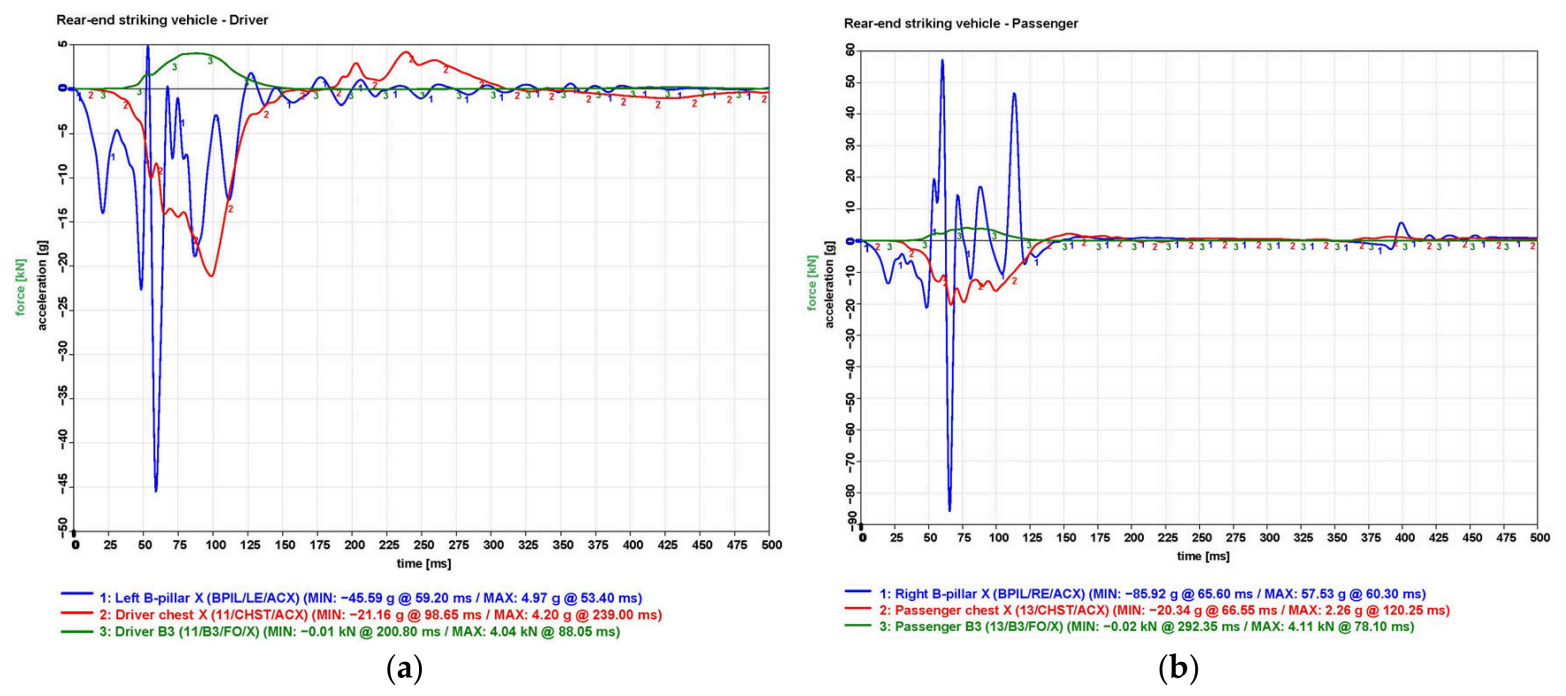
Synchronized multi-sensor responses for the rear-end striking vehicle: (**a**) driver position (11); (**b**) front passenger position (13). Longitudinal B-pillar acceleration, thoracic acceleration, and local B3 seat belt webbing tension are shown relative to t_0_.

**Figure 21:**
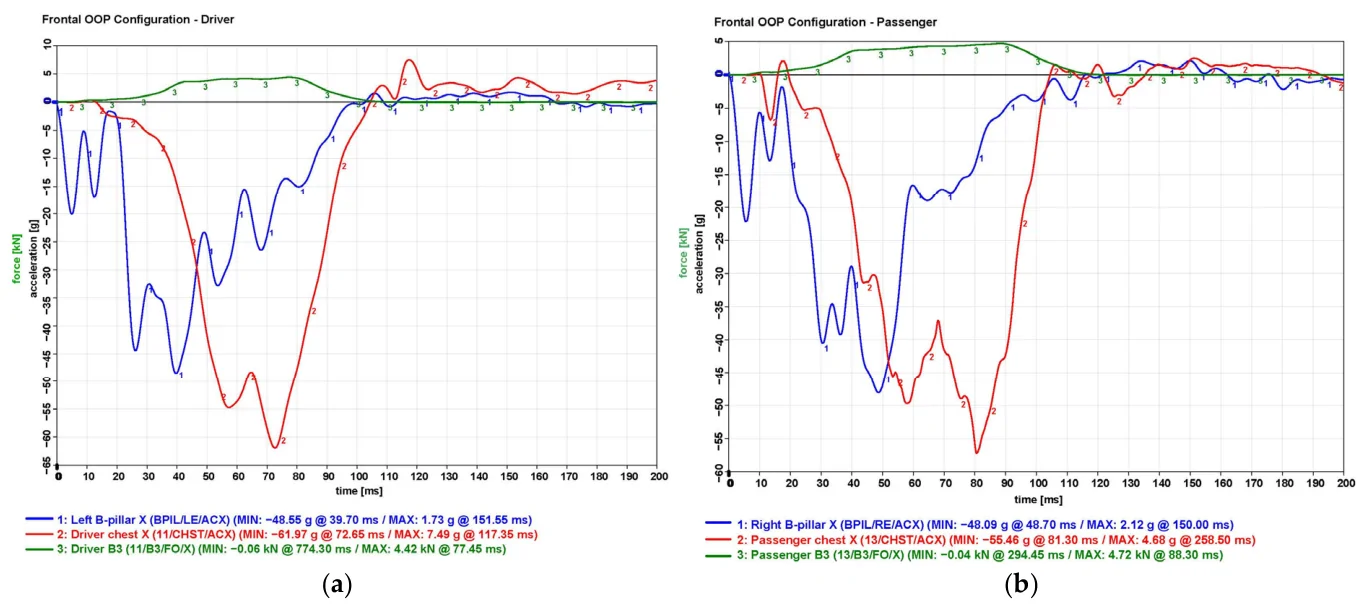
Synchronized multi-sensor responses for the frontal OOP configuration: (**a**) driver position (11); (**b**) front passenger position (13). Longitudinal B-pillar acceleration, thoracic acceleration, and local B3 seat belt webbing tension are shown relative to t_0_.

The comparison included the rear-end target vehicle, the rear-end striking vehicle, and the frontal rigid-barrier configuration with forward-leaning OOP occupants. All measurement channels were referenced to the common impact time (t_0_) defined by the physical contact trigger, as described in Section 2.5. Figure 19, Figure 20 and Figure 21 present the synchronized responses separately for the driver (position 11) and front passenger (position 13).

For the rear-end target vehicle (Figure 19), the synchronized traces show a prolonged response compared with the other investigated configurations. The occupant initially loads the seatback, while appreciable belt loading develops later during the rebound phase. The B5 and B3 belt-force maxima occur at 219.25 ms and 257.20 ms, respectively, while the local B-pillar response reaches its maximum magnitude at 291.85 ms. The B-pillar time history remains active over the later stage of the vehicle-to-vehicle interaction; as confirmed by the synchronized high-speed video in Figure 10b, the vehicles are still in physical contact at the time of the maximum local B-pillar response.

For the rear-end striking vehicle (Figure 20), the structural, restraint, and occupant responses are concentrated within a shorter interval after t_0_. The local B-pillar acceleration rises rapidly after impact and reaches its maximum magnitude at 65.60 ms. The B3 webbing tension reaches its maximum at 78.10 ms, while the maximum longitudinal thoracic acceleration occurs at 98.65 ms. The synchronized traces therefore show overlapping structural, restraint, and occupant responses, with different temporal development and peak times within the same impact event.

For the frontal OOP configuration (Figure 21), the three measurement domains are concentrated within the initial phase following barrier contact. The local B-pillar acceleration reaches its maximum magnitude at 39.70 ms, followed by substantial restraint and occupant loading. The maximum longitudinal thoracic acceleration occurs at 72.65 ms, while the B3 webbing tension shown in the synchronized comparison reaches its maximum at 88.30 ms. The traces show substantial temporal overlap between the structural, restraint, and thoracic responses during the principal occupant-loading interval.

Because the measured signals represent different physical quantities and local transient responses, these temporal characteristics are evaluated descriptively rather than as evidence of physical coupling or causality. Differences between individual peak times are not interpreted as propagation or load-transfer delays. Furthermore, because the number and location of instrumented belt segments differed between configurations, the belt-force channels are considered as local webbing tensions and are not combined into a summed restraint-force quantity.

The combined measurement approach provides synchronized experimental data that may support future development and evaluation of computational occupant-response models. However, the present dataset, comprising one full-scale crash event per investigated configuration, is not intended for model identification or parameter estimation. Such applications require a broader experimental dataset covering multiple impact severities, occupant postures, and restraint conditions, together with independent validation.

## 5. Discussion

The present study provides a detailed analysis of occupant and vehicle responses under different crash scenarios using a synchronized multi-sensor framework. The comparison between the rear-end and frontal configurations demonstrates configuration-specific differences in local structural response, restraint loading, and occupant biomechanical response.

The B-pillar acceleration profiles reveal substantial differences between the test configurations. The rear-impacted vehicle exhibited the highest local peak acceleration (112.30 g). In contrast, lower local B-pillar peak accelerations were recorded for the striking vehicle in the rear-end collision and for the vehicle in the full-frontal rigid-barrier test (85.92 g and 48.55 g, respectively). These differences characterize the local structural response at the instrumented B-pillar locations under the investigated impact conditions and should not be interpreted as whole-vehicle deceleration.

Thoracic acceleration measurements showed distinct response levels across the investigated configurations. In the rear-end collision, the target vehicle exhibited a peak longitudinal (*X*-axis) thoracic acceleration of 12.52 g, while the striking vehicle reached 21.16 g. The frontal OOP configuration exhibited the highest peak longitudinal (*X*-axis) thoracic acceleration, at 61.97 g. These differences are reported as configuration-specific observations and should not be interpreted as isolating the effect of occupant posture, since the investigated cases differed in impact configuration as well as occupant positioning.

For the frontal OOP configuration, selected injury metrics were calculated and are presented in Appendix B. The measured HIC15 and 3 ms resultant thoracic acceleration values are compared descriptively with the corresponding FMVSS 208 [32] reference values for the belted in-position Hybrid III 50th percentile male. However, because the investigated ATDs were intentionally positioned in a forward-leaning posture, this comparison is provided only as contextual information and should not be interpreted as a regulatory compliance assessment or as establishing an equivalent injury-risk interpretation for the OOP condition.

When considered together with the synchronized structural, restraint, and occupant measurements, these injury metrics provide complementary information on the response observed during the frontal OOP test. However, because the study did not include a matched in-position frontal rigid-barrier test under otherwise identical conditions, the differences observed across the investigated configurations cannot be attributed exclusively to occupant posture.

Seat belt force measurements provide additional information on local restraint loading within each configuration. Moderate belt loads were recorded in the rear-end tests, whereas higher local webbing tensions were observed in the frontal OOP configuration. In this configuration, the highest peak was recorded at B6 (outer lap belt), reaching 9.80 kN, followed by B4 and B5, while B3 (upper diagonal belt) exhibited the lowest peak value among the four instrumented segments. This pattern indicates a non-uniform distribution of local webbing loading in the forward-leaning occupant configuration. Because the individual transducers measure tension at specific belt segments, the observed distribution characterizes the local loading of the diagonal and lap portions of the restraint system.

An additional observation concerns the B3 upper diagonal belt segment in the frontal OOP configuration, which reached similar peak values of 4.42 kN and 4.72 kN at positions 11 and 13, respectively, whereas the measured B4–B6 peak values ranged from 5.37 to 9.80 kN. The similar B3 peak levels at the two independently instrumented seating positions may be consistent with the action of a retractor load limiter reaching a comparable force plateau. This interpretation could also contribute to explaining the higher local B4 forces relative to B3. However, because the load-limiter characteristics and limiting levels of the production restraint systems were not available, this remains a candidate explanation rather than a verified restraint-system mechanism.

The synchronized use of structural accelerometers, seat belt force transducers, and ATD instrumentation enables the different measurement domains to be examined within a common temporal reference. This combined approach provides complementary information on local vehicle structural response, restraint loading, and occupant biomechanical response that would not be available from any of these measurement domains considered in isolation. The descriptive time-history analysis further shows that the maxima of the measured responses do not necessarily occur in the same temporal sequence across the investigated configurations; accordingly, differences in peak timing are not interpreted as physical propagation or load-transfer delays.

Overall, the experimental results demonstrate the applicability of the proposed multi-sensor framework for synchronized characterization of occupant–vehicle interaction in controlled full-scale crash tests. The methodology provides a consistent basis for comparing structural, restraint-system, and occupant measurements across different experimental configurations. Such synchronized datasets may also support future development and evaluation of computational occupant models and restraint-system investigations, provided that broader experimental datasets are available for model calibration and independent validation.

The generalizability of the present results is limited by the specific experimental conditions considered in this study. As each experimental configuration was represented by a single full-scale crash event, the observed differences should be interpreted as configuration-specific experimental observations rather than statistically generalizable effects. Test-to-test variability is a recognized consideration in full-scale crash testing [33]; however, it cannot be quantified from the present dataset and should therefore be considered when interpreting comparisons between the investigated configurations. The crash tests were performed using Hybrid III 50th percentile male ATDs and focused on two impact configurations: a rear-end vehicle collision and a full-frontal rigid-barrier impact with an out-of-position occupant posture. Although these scenarios represent relevant crash conditions, the observed responses may vary for different occupant sizes, vehicle structures, impact velocities, or seating configurations. In addition, the analysis was limited to selected measurement locations and parameters, primarily focusing on thoracic acceleration, seat belt forces, and B-pillar structural response. Other injury metrics and body regions may provide complementary insights into occupant behavior under similar conditions.

Nevertheless, the proposed multi-sensor measurement framework is not restricted to the specific scenarios investigated in this study and can be extended to a wide range of experimental configurations. While the numerical results reported here are specific to the analyzed tests, the methodology itself provides a consistent basis for broader investigations of occupant–vehicle interaction in future crash safety research.

Although the experimental campaign was conducted using earlier-generation production vehicles, the proposed measurement approach is not vehicle-specific. The instrumentation, synchronization, and signal-processing methodology can also be applied to current-generation internal combustion, hybrid, and electric vehicles. Therefore, the numerical results reported in this study characterize the investigated test configurations, whereas the experimental approach itself is applicable to a broader range of vehicle platforms.

## 6. Conclusions

The proposed synchronized multi-sensor framework extends conventional crash-test assessment by integrating local vehicle structural acceleration, restraint-system loading, and occupant biomechanical measurements within a common temporal reference. The methodology enables these measurement domains to be evaluated simultaneously and provides a consistent experimental approach for characterizing occupant–vehicle interaction across different crash configurations.

The experimental results demonstrated distinct configuration-specific responses. The rear-impacted vehicle exhibited the highest local B-pillar acceleration, whereas the frontal rigid-barrier configuration with forward-leaning OOP occupants produced the highest longitudinal thoracic acceleration and local belt-segment force. Because the investigated configurations differed in both impact conditions and occupant positioning, these observations should not be interpreted as isolating the effect of occupant posture.

Analysis of the synchronized time histories further showed that structural, restraint, and occupant response maxima did not necessarily occur in the same temporal sequence across the investigated configurations. These temporal characteristics were therefore interpreted descriptively rather than as physical propagation or load-transfer delays. The seat belt measurements similarly represent local webbing tensions at the instrumented belt segments and should not be interpreted as a resultant restraint force or as loads acting directly on specific anatomical regions.

Overall, the study demonstrates the applicability of the proposed framework for synchronized multi-domain characterization in full-scale crash testing. While the numerical findings are specific to the investigated test conditions and cannot be generalized statistically from the present dataset, the methodology provides a reusable experimental basis for broader occupant–vehicle interaction studies.

Future work should extend the experimental dataset to additional impact severities, occupant postures, restraint conditions, and occupant characteristics. Such datasets may support the development and evaluation of computational occupant-response models and advanced restraint-system concepts.

## Figures and Tables

**Table 1:** General characteristics of the vehicles used in the experimental campaign.

Impact Configuration	Vehicle Role	Approx. Model-Year Range	Vehicle Class	Body Style	Front Restraint-System Characteristics
Front–rear-end impact	Target vehicle	2000–2002	Mid-size passenger car	4-door sedan	Three-point seat belts with pretensioners and load limiters; frontal airbags
Front–rear-end impact	Striking vehicle	2000–2001	Small passenger car	5-door hatchback	Three-point seat belts with pretensioners and load limiters; frontal airbags
Full-frontal rigid-barrier impact	Test vehicle	2001–2004	Compact passenger car	5-door station wagon	Three-point seat belts with pretensioners and load limiters; frontal airbags

**Table 2:** Summary of the maximum recorded structural, occupant, and restraint responses across the investigated crash configurations.

Category	Parameter	Rear-End Target	Rear-End Striking	Frontal OOP
Vehicle response	Peak B-pillar acceleration magnitude (g)	112.30	85.92	48.55
	Time to peak (ms)	291.85	65.60	39.70
Occupant response	Peak longitudinal thoracic acceleration magnitude, *X*-axis (g)	12.52	21.16	61.97
	Time to peak (ms)	90.65	98.65	72.65
Restraint system	B3 peak force (kN)/time (ms)	0.99/257.20	4.11/78.10	4.72/88.30
	B4 peak force (kN)/time (ms)	—	2.97/89.85	8.71/59.35
	B5 peak force (kN)/time (ms)	0.58/219.25	—	8.16/56.45
	B6 peak force (kN)/time (ms)	—	2.54/91.10	9.80/56.35

## Data Availability

The processed results supporting the findings of this study, including the reported quantitative values and representative time-history responses, are presented in the article. The complete experimental time-history dataset is not publicly deposited, as it comprises additional measurement data beyond the scope of the present methodological study and may support further dedicated investigations.

## Abbreviations

The following abbreviations are used in this manuscript:

11	ISO-MME Driver seating position
13	ISO-MME Front passenger seating position
A3ms Chest	3 ms Chest Acceleration Criterion
ATD	Anthropomorphic Test Device
B3	Upper diagonal belt measurement position
B4	Lower diagonal belt measurement position
B5	Inner lap belt force measurement position
B6	Outer lap belt force measurement position
CFC	Channel Frequency Class
DAQ	Data Acquisition System
FMVSS 208	Federal Motor Vehicle Safety Standard No. 208
HIC	Head Injury Criterion
ISO	International Organization for Standardization
OOP	Out-of-Position
SAE	SAE International
THOR	Test Device for Human Occupant Restraint
11/CHST/ACX	Chest acceleration in X direction for driver position
11/CHST/ACY	Chest acceleration in Y direction for driver position
11/CHST/ACZ	Chest acceleration in Z direction for driver position
13/CHST/ACX	Chest acceleration in X direction for front passenger position
13/CHST/ACY	Chest acceleration in Y direction for front passenger position
13/CHST/ACZ	Chest acceleration in Z direction for front passenger position
BPIL/RI/AC	Right B-pillar acceleration
BPIL/LE/AC	Left B-pillar acceleration
SEBE	Seat Belt
11/B3/FO/X	Upper diagonal belt webbing tension, driver position
11/B4/FO/X	Lower diagonal belt webbing tension, driver position
11/B5/FO/X	Inner lap belt webbing tension, driver position
11/B6/FO/X	Outer lap belt webbing tension, driver position
13/B3/FO/X	Upper diagonal belt webbing tension, front passenger position
13/B4/FO/X	Lower diagonal belt webbing tension, front passenger position
13/B5/FO/X	Inner lap belt webbing tension, front passenger position
13/B6/FO/X	Outer lap belt webbing tension, front passenger position

## Appendix A

**Table A1:** Technical specifications of the sensors associated with the measurements analyzed in this study.

Sensor Type	Manufacturer	Model	Measuring Range/Rated Capacity	Sensitivity/Output	Calibration	Calibration Interval
**Head accelerometer**	Integrated in Hybrid III 50th percentile ATD	Standard ATD instrumentation	According to Hybrid III specifications		Calibrated and managed under the laboratory quality system according to DIN EN ISO/IEC 17025:2018	12 months
**Chest accelerometer**	Integrated in Hybrid III 50th percentile ATD	Standard ATD instrumentation	According to Hybrid III specifications			12 months
**B-pillar triaxial accelerometer**	Advanced International Sensors	AIS 89C2	±2000 m/s^2^ (≈±204 g)	0.08130 mV/g	Calibration certificate date: 10 July 2024	12 months
**Seat belt force sensor**	ME-Messsysteme GmbH	5BCD1622	16 kN	2.2579 mV/16 kN/1 V	Calibration certificate date: 15 July 2024	12 months

## Appendix B

The injury metrics presented in this appendix correspond exclusively to the full-frontal rigid-barrier test with forward-leaning OOP occupants. The measured HIC15 and 3 ms resultant thoracic acceleration values are compared descriptively with the corresponding injury reference values used for the belted in-position Hybrid III 50th percentile male under FMVSS 208. This comparison is provided for contextual purposes only and does not constitute an FMVSS 208 compliance assessment. Because these reference values were established for nominal occupant positioning, their applicability to the forward-leaning OOP posture investigated here is uncertain, particularly given the limited established biofidelity of the Hybrid III ATD for such non-standard postures.

**Table A2:** Measured Injury Metrics for the Frontal OOP Configuration Compared with FMVSS 208 Reference Values.

Position	Region/Metric	Measured Value	Unit	FMVSS 208 Reference Value	Filter	Interval (ms)
11—Driver	HIC15	775	–	700	CFC 1000	0–300
11—Driver	A3ms Chest (resultant)	62.05	g	60	CFC 180	0–300
13—Front passenger	HIC15	619	–	700	CFC 1000	0–300
13—Front passenger	A3ms Chest (resultant)	57.31	g	60	CFC 180	0–300
